# CD4^+^ T cells expressing CX3CR1, GPR56, with variable CD57 are associated with cardiometabolic diseases in persons with HIV

**DOI:** 10.3389/fimmu.2023.1099356

**Published:** 2023-02-14

**Authors:** Celestine N. Wanjalla, Curtis L. Gabriel, Hubaida Fuseini, Samuel S. Bailin, Mona Mashayekhi, Joshua Simmons, Christopher M. Warren, David R. Glass, Jared Oakes, Rama Gangula, Erin Wilfong, Stephen Priest, Tecla Temu, Evan W. Newell, Suman Pakala, Spyros A. Kalams, Sara Gianella, David Smith, David G. Harrison, Simon A. Mallal, John R. Koethe

**Affiliations:** ^1^Division of Infectious Diseases, Vanderbilt University Medical Center, Nashville, TN, United States; ^2^Division of Gastroenterology, Vanderbilt University Medical Center, Nashville, TN, United States; ^3^Division of Endocrinology, Vanderbilt University Medical Center, Nashville, TN, United States; ^4^Vaccine and Infectious Diseases Division, Fred Hutchinson Cancer Center, Seattle, WA, United States; ^5^Division of Rheumatology, Vanderbilt University Medical Center, Nashville, TN, United States; ^6^Division of Allergy, Pulmonary and Critical Care Medicine, Vanderbilt University Medical Center, Nashville, TN, United States; ^7^Department of Global Health, University of Washington, Seattle, WA, United States; ^8^Department of Medicine, University of California, San Diego, CA, United States; ^9^Division of Clinical Pharmacology, Vanderbilt University Medical Center, Nashville, TN, United States; ^10^Infectious Disease Section, Veterans Affairs Tennessee Valley Healthcare System, Nashville, TN, United States

**Keywords:** HIV, CD4 T cells, cardiometabolic disease, cytomegalovirus, CGC

## Abstract

Persons with HIV (PWH) on long-term antiretroviral therapy (ART) have a higher incidence and prevalence of cardiometabolic diseases attributed, in part, to persistent inflammation despite viral suppression. In addition to traditional risk factors, immune responses to co-infections such as cytomegalovirus (CMV) may play an unappreciated role in cardiometabolic comorbidities and offer new potential therapeutic targets in a subgroup of individuals. We assessed the relationship of CX3CR1^+^, GPR56^+^, and CD57^+/-^ T cells (termed CGC^+^) with comorbid conditions in a cohort of 134 PWH co-infected with CMV on long-term ART. We found that PWH with cardiometabolic diseases (non-alcoholic fatty liver disease, calcified coronary arteries, or diabetes) had higher circulating CGC^+^CD4^+^ T cells compared to metabolically healthy PWH. The traditional risk factor most correlated with CGC^+^CD4^+^ T cell frequency was fasting blood glucose, as well as starch/sucrose metabolites. While unstimulated CGC^+^CD4^+^ T cells, like other memory T cells, depend on oxidative phosphorylation for energy, they exhibited higher expression of carnitine palmitoyl transferase 1A compared to other CD4^+^ T cell subsets, suggesting a potentially greater capacity for fatty acid β-oxidation. Lastly, we show that CMV-specific T cells against multiple viral epitopes are predominantly CGC^+^. Together, this study suggests that among PWH, CGC^+^ CD4^+^ T cells are frequently CMV-specific and are associated with diabetes, coronary arterial calcium, and non-alcoholic fatty liver disease. Future studies should assess whether anti-CMV therapies could reduce cardiometabolic disease risk in some individuals.

## Introduction

Persons with HIV (PWH) are at increased risk of cardiovascular and metabolic diseases compared to the general population (1), which has been attributed to persistent systemic inflammation despite the effective suppression of plasma viremia on antiretroviral therapy (ART) (2–4). Among PWH, co-infection with other viruses, such as cytomegalovirus (CMV) and hepatitis B and C, increases the risk of diabetes, cardiovascular disease, and cerebrovascular events, as well as other non-AIDS illnesses (5–8). The adaptive immune system devotes a relatively large proportion of memory CD4^+^ and CD8^+^ T cells to the anti-CMV response as compared to other viruses (9–12), and this disproportionate inflation is further exaggerated in PWH compared to age-matched individuals without HIV (10, 13). As such, HIV presents an important natural model to investigate how sustained exposure to CMV affects various aspects of the immune response and contributes to other aging-related disease processes.

CMV is a herpesvirus that co-evolved with mammals and infects many individuals at a young age, and CMV is highly prevalent in some groups including adult PWH (14, 15). Growing evidence suggests that anti-CMV T cell responses have a role in metabolic dysregulation in both animal models (16) and humans (17–22). CMV-seropositivity has been shown to predict severe non-AIDS-related illnesses and is an independent risk factor for cardiovascular and cerebrovascular disease in PWH (8, 23, 24). In PWH, the high prevalence of CMV is such that CMV-seropositivity alone does not stratify individuals at risk of developing these comorbidities. However CMV antibody titers were not associated with cardiovascular mortality in HIV-negative community-dwelling adults, suggesting antibody titers alone may not fully reflect the impact of anti-CMV immune responses on disease risk. (25).

Prior studies from our group suggest that an increase in CMV-specific CD4^+^ T cells in PWH may serve as a novel marker of metabolic and cardiovascular disease risk. We previously showed that adipose tissue CD4^+^ T cells co-expressing CX3CR1, GPR56, and CD57 (termed ‘CGC^+^’ cells), a surface marker combination suggestive of antiviral activity, increased with progressive glucose intolerance (26) and with carotid plaque burden in PWH (18). Furthermore, we showed that CMV-specific CD4^+^ T cells that recognized an immunodominant peptide epitope (DYSNTHSTRYV from glycoprotein B (gB)) restricted through HLA-DR7 were predominantly CGC^+^ (17, 18). Further, we demonstrated that CGC^+^ CD4^+^ T cells were cytotoxic and oligoclonal (17). Despite several studies describing a role for CMV-seropositivity in morbidity and mortality in PWH (8), little is known about the role of CMV-specific CD4^+^ and CD8^+^ T cells, target viral epitopes, and the basic mechanisms that mediate these pathologies.

A study of pneumococcal vaccine responses in persons with antineutrophil cytoplasmic antibody-associated vasculitis showed that treatment with valacyclovir reduced subclinical CMV and the proportion of CD4^+^ CD28^-^ T cells, and improved responses to the vaccine (27). The expansion of CD4^+^ CD28^-^ T cells was thought to have reduced the functional capacity of the CD4^+^ T cell memory compartment, and thereby reduced responses to vaccines. Although valacyclovir is not a recommended first-line therapy against CMV, this study suggests the possible role of anti-CMV therapy in reducing the proportion of CD4^+^ CD28^-^ T cells, a population with considerable overlap with CGC^+^ CD4^+^ T cells (26). A similar study in PWH showed that treatment with valganciclovir reduced detectable CMV DNA levels and reduced CD8^+^ T cell activation, defined by CD38 and HLA-DR expression, at 8 weeks and 12 weeks of treatment (28). Notably, there was no difference in soluble inflammation biomarkers between the placebo group and those treated with valganciclovir, suggesting this effect was primarily on circulating T cells. These findings are important clinically, as they suggest that the anti-CMV T cell response is malleable, and if CD4^+^ CD28^-^ or CGC^+^ T cells contribute to cardiometabolic disease pathogenesis the use of anti-viral agents could serve as a novel therapeutic strategy.

The goal of the current study was to (1) characterize the CMV specificity of CGC^+^ cells using a broader range of tetramer staining, (2) evaluate the relationship between peripheral blood CGC^+^ T cells with cardiovascular and metabolic diseases among a large cohort of PWH on ART, and (3) determine the relationships between CGC^+^ T cells and traditional cardiovascular disease risk factors. Using a wide array of class I and II CMV tetramers, we show that CMV-specific T cells are CX3CR1^+^ and GPR56^+^ with variable expression of CD57, and are among the cluster defined as CGC^+^. We show that circulating CGC^+^ CD4^+^ T cells and CGC^+^ CD8^+^ T cells in PWH are associated with prevalent cardiometabolic conditions (diabetes, subclinical atherosclerosis, and liver disease). Among individual disease risk factors, CGC^+^ T cells are related most strongly to fasting blood glucose and hemoglobin A1C. CGC^+^ T cells are also correlated with starch and sucrose metabolites measured in the plasma of PWH. Notably, total memory CD4^+^ and CD8^+^ T cells do not have a similar relationship with starch and sucrose metabolites. The relationship between circulating CGC^+^ T cells and fasting blood glucose does not appear to be driven by a greater dependence on glucose metabolism as a source of energy when compared to other memory T cell subsets. However, higher expression of carnitine palmitoyl transferase 1A (CPT1A) by CGC^+^ T cells may be due to a greater capacity for fatty acid β-oxidation CPT1A. These findings suggest that CGC^+^ T cell expansion associated with cardiometabolic disease in PWH may be driven by CMV co-infection. Additional prospective studies defining the antigen specificity of CGC^+^ T cells and their mechanistic role in cardiometabolic disease pathogenesis are underway.

## Methods

### Study participants

From August 2017 and November 2019, we recruited 134 adults PWH without diabetes (fasting blood glucose (FBG) <100 mg/dl and/or hemoglobin A1c (HbA1c) <5.7%), with pre-diabetes (FBG 100-125 mg/dl and/or HbA1c 5.7-6.4%) or with diabetes (FBG ≥ 126 mg/dl and/or HbA1c ≥6.5% or on anti-diabetic medications) to the *HIV, Adipose Tissue Immunology and Metabolism* (HATIM) study from the Vanderbilt Comprehensive Care Clinic, an academic, urban HIV treatment facility (17). All participants were on ART combination therapy for ≥18 months, with a minimum of 12 months of sustained plasma viral suppression, a CD4^+^ T cell count >350 cells/μl, and no known inflammatory or rheumatologic conditions. Exclusion criteria were self-reported heavy alcohol use (>11 drinks/week), known liver cirrhosis, active hepatitis B or C, cocaine or amphetamine use, and use of corticosteroids or growth hormones. Anthropometric measurements including waist circumference, height, weight, and body mass index (BMI) were obtained on the day of recruitment (**Table 1**). Diabetic PWH were older, with significantly fewer smokers. Participants provided written informed consent, and the study was approved by the Vanderbilt University Institutional Review Board. The study is registered at ClinicalTrials.gov (NCT04451980).

**Table 1:** Clinical and demographic characteristics of the study cohort.

	N	Non-Diabetic PWH N=51	Pre-Diabetic PWH N=44	Diabetic PWH N=39	Test Statistic
Age, yrs	134	45 [36, 52]	44 [36, 56]	54 [49, 58]	**0.001**
Sex, male	134	0.80 ^41^/_ 51_	0.80 ^35^/_ 44_	0.72 ^28^/_ 39_	0.6
Race, Caucasian	134	0.59 ^30^/_ 51_	0.52 ^23^/_ 44_	0.46 ^18^/_ 39_	0.5
Smoker status, yes	131	0.36 ^18^/_ 50_	0.30 ^13^/_ 44_	0.11 ^4^/_ 37_	**0.001**
Hepatitis C ab status	134	0.20 ^10^/_ 51_	0.09 ^4^/_ 44_	0.13 ^5^/_ 39_	0.3
HTN, yes	134	0.63 ^32^/_ 51_	0.57 ^25^/_ 44_	0.67 ^26^/_ 39_	0.9
BMI (Kg/m^2^)	134	30.7 [28.1, 34.1]	31.8 [29.0, 35.3]	33.8 [30.3, 39.2]	**0.01**
Waist circumference (cm)	132	104 [92, 109]	106 [95, 113]	112 [107, 120]	**0.001**
Laboratory values					
Hemoglobin A1C, %	132	5.3 [4.9, 5.4]	5.6 [5.2, 5.9]	6.8 [6.2, 8.9]	**<0.001**
FBG, mg/dL	131	90 [83, 94]	111 [104, 118]	161 [128, 234]	**0.001**
Creatinine, mg/dL	132	1.0 [0.9, 1.1]	1.0 [0.8,1.1]	1.0 [0.9, 1.3]	0.3
LDL, mg/dL	131	96 [84, 120]	110 [93, 127]	90 [80, 105]	**0.03**
Cholesterol, mg/dL	133	170 [149, 202]	180 [166, 212]	175 [150, 196]	0.3
HDL, mg/dL	133	44 [36, 54]	41 [34, 50]	40 [34, 46]	0.5
Triglycerides, mg/dL	133	104 [77, 170]	128 [90, 196]	165 [114, 262]	**0.006**
HsCRP, mg/dL	131	2.7 [1.2, 5.1]	2.7 [1.1, 4.1]	3.0 [2.1, 7.7]	0.2
Statin use, yes	124	0.22 ^10^/_ 45_	0.32 ^14^/_ 44_	0.63 ^22^/_ 35_	**<0.001**
Non-contrast CT imaging					
Pericardial fat, cm3	112	55 [35,80]	75 [45,108]	91 [64,202]	**0.008**
Visceral fat, cm3	113	143 [90, 163]	169 [120, 215]	200 [130, 280]	**<0.001**
Liver mean density, hu	112	63.0 [58.6, 65.6]	62.3 [55.0, 67.2]	60.5 [46.2, 63.0]	**0.04**
CAC prevalence, yes	113	0.02 ^1^/_ 41_	0.33 ^12^/_ 36_	0.44 ^16^/_ 36_	**<0.001**
HIV-related Laboratory Values					
CD4 at ART start, cells/ml	130	508 [342, 652]	424 [310, 554]	462 [249, 620]	0.6
CD4 T count at enrollment, cells/ml	134	799 [596, 942]	832 [627, 1016]	945 [732, 1154]	0.08
CD4 cell percentage	134	37.0 [31.5, 41.0]	34.5 [28.8, 38.5]	40.0 [35.0, 45.5]	**0.004**
Duration ART, yrs	131	6.7 [4.3, 11.6]	7.1 [3.1, 11.2]	8.9 [5.0, 16.2]	0.2

N is the number of non-missing values.  Statistical tests used: Kruskal-Wallis test for continuous variables; Pearson chi-square test for categorical variable. Bold values indicate p-values <0.05.

### Sample collection

Subcutaneous adipose tissue was obtained from participants by liposuction and the stromal vascular fraction (SVF) was processed within 30 minutes to 1 hour of the procedure as previously published in detail (17). Peripheral blood mononuclear cells (PBMCs) were processed by Ficoll gradient. PBMCs and SVF from all participants were cryopreserved and subsequent assays were performed at a later date in batches. We also re-analyzed data single-cell metabolic profiling of T cells obtained from healthy human donors at the Stanford Blood Center, according to the guidelines of the Stanford Institutional Review Board (29).

### Computed tomography imaging

We performed non-contrast computed tomography (CT) imaging within 1 week of blood collection and anthropometric measurements. This was performed using a Siemens Somatom Force multidetector scanner (Erlangen, Germany). Total coronary arterial calcium (Agatston units, Au) was measured in the left anterior descending (LAD), left main (LM), left circumflex (LCX), and right coronary artery (RCA). For our analysis, coronary arterial calcium (CAC) was treated as a categorical variable (presence or absence of coronary CAC). The mean coronary cross-sectional area (external diameter of the outer walls) was measured at three equidistant points of the LAD. The mean coronary cross-sectional area (corCSA) was derived from the mean of three points and used as a surrogate for arterial remodeling. Non-alcoholic fatty liver disease was defined by liver attenuation, which was averaged from nine total regions using the open-source OsiriX software field (30). Perivascular adipose tissue (PAT) volume (adipose tissue around the LM coronary, LAD, circumflex, and RCA) and epicardial adipose were measured as previously described (30).

### Flow cytometry

A multiparameter flow cytometry antibody panel was used to stain PBMCs (17, 18, 26). The panel used to define CGC^+^ cells included anti-CD3, CD4, CD8, CCR7, CD45RO, GPR56, CX3CR1, CD57, CD14, CD19, and LIVE/DEAD Aqua (**Supplemental Table 1**). We included Class I and Class II CMV tetramers with this panel to identify virus-specific T cells. CMV Class I (pp65 [HLA-A02 NLVPMVATV (NLV)]) and Class II tetramers (gB [HLA DR1:07 DYSNTHSTRYV (DYS), pp65 [HLA DR1:03 EFFWDANDIYRIF (EFF)], IE2 [HLA DR1:03 TRRGRVKIDEVSRMF (TRR), IE1 [HLA DR1:03 VKQIKVRVDMVRHRI (VKQ)] and pp65 [HLADQB1*06:02 LLQTGIHVRVSQPSL (LLQ)] were obtained from the NIH tetramer facility supported by contract 75N93020D00005 from the National Institute of Allergy and Infectious Diseases. The analysis was performed using the BD FACS Aria II flow cytometer. Bulk and single-cell sorting was performed using a 70μm nozzle into 96 well plates or Eppendorf tubes, respectively, as previously published (17). An additional panel with fluorescently tagged antibodies was used to further characterize KLRG1, CD27, and CD28 expression on CGC^+^ T cells (anti-GPR56, CCR7, CD38, KLRG1, CD14, CX3CR1, CD45RO, CXCR3, PD1, CD27, CD57, CD3, Live/Dead stan, CD8, CD4, CD28, and CXCR5) (**Supplemental Table 1**). These samples were run on a Cytek/Aurora. We used Cytobank to analyze the flow cytometry and mass cytometry data (31). The gating strategy used to define the immune subsets is shown in the **Supplemental Material** (**Supplemental Figure 1**).

### Metabolomics sample extraction

Plasma samples were aliquoted at 25 µl and spiked with 5 µL of metabolomics internal standards solution. Extraction of metabolites was performed by protein precipitation by adding 200 µL of 8:1:1 Acetonitrile: Methanol: Acetone (Fisher Scientific, San Jose, CA) to each sample. Samples were mixed thoroughly, incubated at 4°C for 30 min to allow protein precipitation, and centrifuged at 20,000xg to pellet the proteins. After centrifugation, 190 µl supernatant was transferred into a clean microcentrifuge tube and dried under a gentle stream of nitrogen at 30°C (Organomation Associates, Inc., Berlin, MA). Samples were reconstituted with 25 µL of injection standards solution, mixed, and incubated at 4°C for 10-15 min. Reconstituted samples were centrifuged at 20,000xg and supernatants were transferred into LC-vials for LC-MS analysis.

### Metabolomics LC-MS analysis

LC-MS untargeted metabolomics was performed on a Thermo Q-Exactive Orbitrap mass spectrometer equipped with a Dionex UPLC system (Thermo, San Jose, CA). Separation was achieved on an ACE 18-pfp 100 x 2.1 mm, 2 µm column (Mac-Mod Analytical, Inc., Chadsford, PA) with mobile phase A as 0.1% formic acid in water and mobile phase B as acetonitrile (Fisher Scientific, San Jose, CA). The gradient was run at a flow rate of 350 µL/min and consisted of: 0-3 min, 0% B; 3-13 min, 80% B, 13-16 min, 80% B, 16-16.5 min, 0% B. The total run time was 20.5 min. The column temperature was set at 25°C. The injection volume was 4 µL for negative and 2 µL for positive polarity. All samples were analyzed in positive and negative heated electrospray ionization with a mass resolution of 35,000 at m/z 200 as separate injections. The heated-electrospray conditions are 350°C capillary temperature, 3.5 kV capillary voltage, 50 sheath, and 10 arbitrary units of auxiliary gas. LC-MS injection was done following a sequence of 3 blanks, neat QC, pooled QC, 10 randomized samples, blank, neat QC, pooled QC, 10 randomized samples, and so on.

### Metabolomics data processing

The percent relative standard deviation of internal standard peak areas was calculated to evaluate extraction and injection reproducibility. The raw files were then converted to mzXML using MS Convert (ProteoWizard, Palo Alto, CA). Mzmine 2 was used to identify features, deisotope, align features and perform a gap filling to fill in any features that may have been missed in the first alignment algorithm. The data were searched against an internal retention time metabolite library. All adducts and complexes were identified and removed from the data set.

### Mass cytometry by time of flight

We used cytometry by time of flight (CyTOF) to further define cell surface markers expressed on CGC^+^ T cells. In brief, cryopreserved PBMCs were thawed and treated with Nuclease S7. After two washes, the cells were stained with LIVE/DEAD Cisplatin stain for 3 minutes, followed by quenching, and then stained with a master mix of CyTOF antibodies against surface markers. Sixteen percent PFA was used to fix cells for 15 minutes at room temperature. After one wash, we resuspended cells in 1mL cold methanol, and caps were sealed with parafilm before incubating overnight at -20°C. On the day the cells were to be analyzed, we washed them with 1X PBS/1% BSA. They were then stained with the intracellular marker CTLA4 for 20 minutes at room temperature. This was followed by staining with a 25μM DNA intercalator (Ir) in the presence of 1.6% PFA for 20 minutes at room temperature and then transferred to 4C until analyzed. Just before analysis on Helios, we washed cells with PBS followed by a wash with Millipore H2O. We resuspended 500,000 cells/ml (in minimum 500uL) ddH2O for the CyTOF run. We added 1/10th volume of equilibration beads to the cells and filtered the cells immediately before running. The Cytof panel included antibodies that define T cells (CD3, CD4, CD8) and memory subsets (CD45RA, CD45RO, CCR7).

For the metabolic profiling, cryopreserved PBMCs from healthy donors were thawed in a cell culture medium (CCM; RPMI 1640 containing 10% FBS, and GlutaMAX; Thermo Fisher Scientific) supplemented with 1:10000x Benzonase (Sigma-Aldrich). Cells from different donors were live cell barcoded using CD45 antibodies as previously described (32), then washed and combined for downstream cell staining. Cells were suspended in TruStain FcX Fc blocker (BioLegend) for 10 min at RT and washed in cell staining media (CSM: PBS with 0.5% BSA and 0.02% sodium azide) before staining. Surface staining was performed in CSM for 30 min at RT. Cells were resuspended in monoisotopic cisplatin-195 for 5 min to label non-viable cells (Fluidigm, 0.5 uM final concentration in PBS). Cells were washed in CSM and fixed and permeabilized using the Foxp3/Transcription Factor Staining Buffer Set (eBiosciences). Intracellular staining was performed in a permeabilization buffer for 30 min at RT. Cells were then washed and resuspended in intercalator solution (1.6% PFA in PBS and 0.5 mM rhodium-intercalator (Fluidigm)) for 1 hr at RT. Cells were washed and resuspended in CSM + 10% DMSO and cryopreserved. Before the acquisition, cells were thawed in CSM and washed twice in Cell Acquisition Solution (CAS; Fluidigm). All samples were filtered through a 35 mm nylon mesh cell strainer, resuspended in CAS supplemented with 1x EQ four-element calibration beads (Fluidigm), and acquired on a Helios mass cytometer (Fluidigm).

### Primary CD4 and CD8 T cell expansion

CGC^+^ and non-CGC^+^ CD4+ and CD8+ T cells were sorted from PBMCs stained with the multiparameter flow cytometry panel. Greater than 90% purity was confirmed by spot checks of sorted cells. The CGC^+^ T cells were expanded using the ImmunoCult™ Human CD3/CD28 T Cell Activator (Stem Cell Technologies, #10991). Cells were expanded per the manufacturer’s protocol with the replacement of media supplemented with human interleukin (IL)-2 (10 to 50ng/ml) every 2-3 days, depending on the density of the cells. Cells were expanded past 14 days and were re-stimulated once more by the addition of CD3/CD28.

### Plasma CMV IgG levels

CMV IgG levels were measured in the plasma by ELISA per the manufacturer’s protocol (Genway, # GWB-BQK12C).

### Single-cell TCR sequencing

Single-cell T cell receptor (TCR) sequencing was performed as published (17). In brief, we stained PBMCs and index-sorted CGC^+^CD4^+^ T cells by flow cytometry into 96- well plates containing 3µL of lysis buffer with a ribonuclease inhibitor (33, 34). We used uniquely tagged primers (TSOend primer and the constant region primers, TCRA or TCRB) for reverse transcription, which tags the cDNA with well-specific barcodes coupled with a unique molecular identifier (UMI) to allow for multiplexing. Samples from each well were then pooled and amplified using the KAPA HiFi HotStart ReadyMix (Roche, Basel, Switzerland). Nested polymerase chain reactions were performed to target the TCR region specifically. We purified the PCR products using Agencourt AMPure XP (Beckman Coulter, CA, UWA) and indexed libraries were created for sequencing using Truseq adapters. The prepared libraries were quantified using the KAPA Universal qPCR Library Quantification Kit (Kapa Biosystems Inc., MA, USA). The products were sequenced on an Illumina MiSeq using a 2×300bp paired-end chemistry kit (Illumina Inc., CA, USA). Reads were quality-filtered and passed through a demultiplexing tool to assign reads to individual wells and mapped to the TCRB and TCRA loci. We used the MIXCR software package to assign TCR clonotypes. We used the visual genomics analysis studio (VGAS), an in-house program for visualizing and analyzing TCR data (http://www.iiid.com.au/software/vgas).

### RNA transcriptomic analysis

Our group previously sorted CGC^+^CD4^+^ and CGC^+^CD8^+^ T cells, as well as other memory T cells, for RNA transcriptomic analysis as published (17). Here, we performed a secondary differential expression analysis to assess differences in 475 genes involved in metabolic pathways between cell types (**Supplemental Table 3**).

### Cytokine assays

We measured interleukin (IL)-4, IL-10, IL-6, and highly sensitive reactive protein (hs-CRP) in plasma using a multiplex assay (Meso Scale Diagnostics, Rockville, MD) as previously published (35).

### Cellular metabolic assays

We analyzed the metabolic profile of CGC^+^CD4^+^ T cells and CGC^+^CD8^+^ T cells *ex vivo* using the SCENITH assay as published (36). In brief, cryopreserved PBMCs were rapidly thawed and resuspended in RPMI media supplemented with 10% fetal bovine serum. After two washes, the cells were stained with antibodies against CCR7 and CX3CR1 at 37°C for 15 minutes. The cells were added in duplicate per condition to 96-well plates at about 1 million cells per well in 180ul R10 media. They were rested for 15-30 minutes at 37°C. 2-Deoxy-D-glucose (2mM), oligomycin (3mM), and DGO (1mM 2DG and 1.5mM oligomycin) were added to the cells, which were incubated at 37°C for 30 minutes. This was followed by the addition of puromycin (10μM) to each well, incubated at 37°C. We included samples without puromycin as controls. The cells were incubated at 37°C for 30 minutes. Cells were spun down and washed with PBS twice. These were then stained with surface antibodies for 15 minutes at room temperature. The cells were washed twice and fixed using the Foxp3/Transcription factor fixation fix/perm solution (20 minutes) and then washed with the Foxp3/Transcription factor permeabilization buffer. This buffer was used to dilute the anti-puromycin antibody. Cells were incubated with the anti-puromycin antibody for 20 minutes at room temperature. Cells were then washed with PBS and immediately analyzed by flow cytometry.

We sorted CGC^+^ and non-CGC^+^ CD4^+^ and CD8^+^ T cells and expanded them as above to obtain enough cells for the Seahorse assay. After 12 days of expansion, 100,000 cells per well were plated on Cell-Tak (Corning) coated plates in Seahorse XF Base Medium (Agilent, 102353-100) supplemented with 1 mM L-glutamine and 1 mM pyruvate at pH 7.4. Seeded cells were centrifuged at 200 g without break and incubated for 1 h at 37° C in a non-CO (2) chamber. ECAR measurements were taken using the Agilent Seahorse XF96 analyzer under basal conditions and after consecutive injections with 10 mM Glucose, 1.5 uM Oligomycin, and 50 mM 2-deoxy-glucose (2-DG). Control wells with assay medium lacking cells were used for background measurements.

### Statistical analysis

Continuous variables/clinical demographics are presented as median values [25^th^ and 75^th^ percentiles], and statistical analyses comparing the three metabolic groups were performed using the Kruskal-Wallis test. Differences between categorical variables, represented as proportions, were analyzed using the Chi-squared test. TCR and RNA transcriptomic analyses were performed using the visual genomics analytics studio tool (VGAS) (37). Differential gene expression between CGC^+^ T cells and non-CGC^+^ T cells was performed using Kruskal Wallis tests, and adjustment for multiple corrections using the Benjamini Hochberg (BH) method. The top differentially expressed genes, p-value < 0.1, were included in the KEGG pathway and Gene Ontology pathway enrichment analysis using Enrichr and Appster (38–40). The relationship between CGC^+^ T cells and plasma metabolites was analyzed using Spearman’s rank correlation. Metabolomic pathways represented by metabolites correlated with CGC^+^ T cells were analyzed using MetaboAnalyst 5.0. Over Representation Analysis (ORA) of the significant plasma metabolites was performed using the hypergeometric test. One-tailed adjusted p values are provided after correcting for multiple testing (FDR). We used the Kruskal-Wallis test to analyze differences in the proportions of immune cell subsets between two groups/treatments, and Wilcoxon tests when there were more than two groups. Relationships between immune subsets and anthropometric or clinical laboratory measurements were determined using Spearman’s rank correlation analysis. We also measured relationships between the immune subsets and other factors after adjustment for potential confounders using partial Spearman’s rank analysis. Statistical analysis was performed using R version 4.1.0 (41) and Prism version 9.

### Data and code availability

The data presented in the study are deposited in the NIH GeneExpression Omnibus repository, accession number GenBank: GSE159759. Differential gene expression was performed using custom software, Visual genomics analysis studio (VGAS) (37).

## Results

### CMV-specific CD8^+^ T cells co-express CX3CR1 and GPR56 with variable CD57

**Figure 1:**
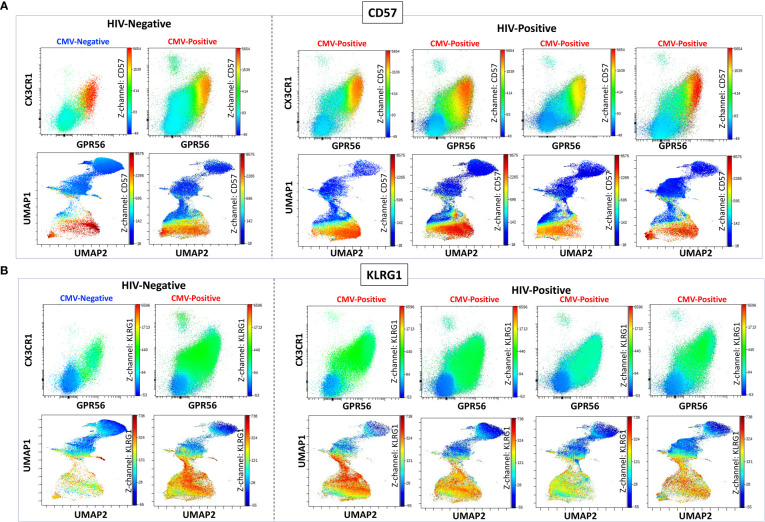
CGC^+^ CD8^+^ T cells express CX3CR1 and GPR56 with variable expression of CD57. The two-dimensional flow cytometry panel shows the co-expression of CX3CR1 and GPR56 and highlights CD57 expression on these cells compared to non-CGC^+^ T cells. UMAP shows CGC^+^ CD8^+^ cluster and variable expression of CD57 **(A)**. KLRG1 expression on CGC^+^ CD8^+^ T cells is demonstrated by two-dimensional plots and UMAP **(B)**. Participants in this analysis included two HIV-negative persons with and without CMV, and four CMV-positive PWH.

We previously defined CMV-specific CD4^+^ T cells that bind the DYSNTHSTRYV epitope (DYS, HLA-DR7, glycoprotein B (gB)) as CGC^+^, however the extent to which this subset of cells is CMV-specific is unknown (18). We characterized CGC^+^ T cells using additional markers that have been associated with CMV-specific T cells. Two-dimensional plots show CX3CR1, GPR56, and CD57 expression on CD8^+^ T cells in two HIV-negative donors (CMV-negative and CMV-positive) and four CMV-positive PWH (**Figure 1A**). We used the Uniform Manifold Approximation and Projection (UMAP) algorithm to visualize clusters of CD8^+^ T cells related by marker expression. The CGC^+^ cluster on CD8^+^ T cells had variable CD57 expression (**Figure 1A**, UMAPs) and expressed the killer-cell lectin-like receptor G1 (KLRG1, a marker associated with senescence) (**Figure 1B**). Additional markers included in the panel defined the CGC^+^ CD8^+^ T cell cluster as largely made up of T effector memory RA-revertant (TEMRA) cells (CD45RO^-^ CCR7^-^), CD28^-^, CD27^+/-^ and CD38^+^ (**Supplemental Figures 2A, B**).

**Figure 2:**
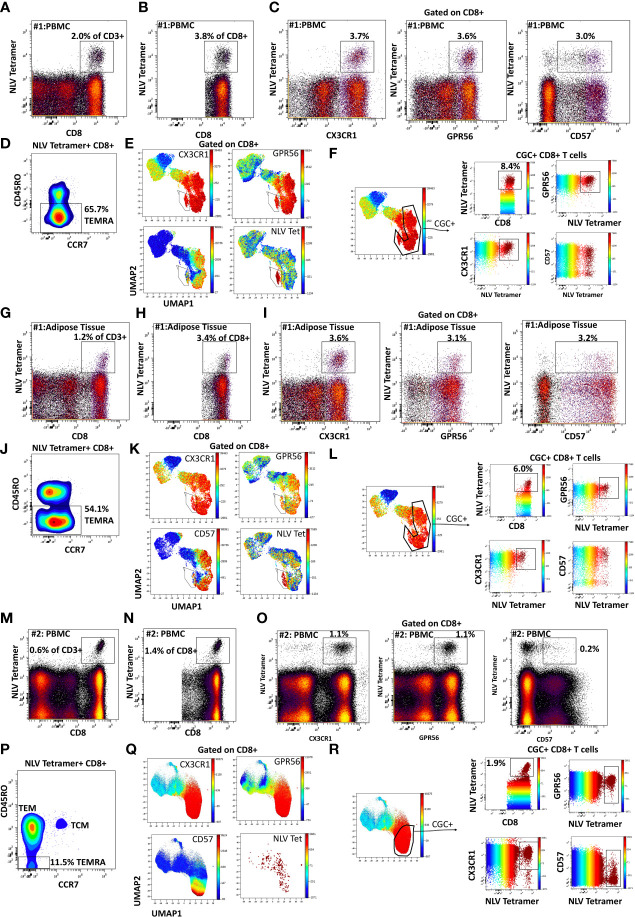
CMV-specific CD8^+^ T cells are predominantly CGC^+^. Phenotypic expression of CX3CR1, GPR56, and CD57 by NLV-specific CD8^+^ T cells in two participants. Peripheral blood NLV-specific CD8^+^ T cells as a proportion of CD3^+^ T cells **(A)** and total CD8^+^ T cells **(B)**. Two-dimensional plots show the co-expression of CX3CR1, GPR56, and CD57 by NLV-specific CD8 T cells **(C)**. Memory cell phenotypes were classified as TEM (CD45RO^+^ CCR7^-^) and TEMRA (CD45RO^-^ CCR7^-^) **(D)**. UMAP of CD8^+^ T cells showing the CD8^+^ T cells with NLV tetramers among the CGC cells **(E, F)**. The proportion of NLV-tetramer^+^ CD8^+^ T cells in matched SVF fraction **(G, H)**. Co-expression of CGC markers **(I)** and memory T cell subsets **(J)** within UMAPs as the proportion of total CGC^+^ CD8^+^ T cells **(K, L)**. A representative sample from the blood of a second participant sample is shown **(M-R)**.

We characterized the CMV-specificity of CD8^+^ T cells from a subset of HLA-typed individuals (**Supplemental Table 2**). In participant #1, we evaluated CMV-specific CD8^+^ T cells in the peripheral blood and adipose tissue using a class I tetramer against the HLA-A*02-01 binding CMV 65 kilodalton phosphoprotein (pp65) epitope_495-503_ NLVPMVATV (NLV). NLV tetramer^+^ cells constituted 2.0% of total CD3^+^ T cells and 3.8% of total CD8^+^ T cells in the peripheral blood of participant #1 (**Figures 2A, B**). Two-dimensional flow cytometry plots show that NLV-tetramer^+^ CD8^+^ T cells co-express CX3CR1 and GPR56 with variable expression of CD57 (**Figure 2C**). 65.7% of the NLV-tetramer^+^ CD8^+^ T cells are TEMRA and the rest effector memory T cells (TEM) (**Figure 2D**). NLV tetramer^+^ cells were present in the cluster of CX3CR1^+^ and GPR56^+^ cells with variable expression of CD57 (**Figure 2E**). We gated on the CGC^+^ CD8^+^ cluster and show the expression of the NLV tetramer, CX3CR1, GPR56 and CD57 (**Figure 2F**). The coloring channel highlights NLV-tetramer^+^ cells (bright red) and shows that 8.4% of the CGC^+^ CD8^+^ T cell cluster from participant #1 express TCRs that bind the NLV tetramer. For this participant #1, we also analyzed NLV tetramer^+^ CD8^+^ T cells in the adipose tissue, given the contribution of adipose tissue inflammation to the development of cardiometabolic disease. 1.2% of total CD3^+^ T cells and 3.4% of total CD8^+^ T cells expressed the NLV TCR (**Figures 2G, H**). These NLV-specific CD8^+^ T cells present in the adipose tissue also co-expressed CX3CR1 and GPR56, with variable CD57^+^ expression. Like the matched peripheral blood, 54.1% of the NLV-tetramer^+^ T cells in adipose were TEMRA and the rest were TEM (**Figures 2I, J**). UMAPs show that NLV-tetramer^+^ CD8^+^ T cells present in the adipose tissue from participant #1 cluster with CGC^+^ CD8^+^ T cells, with a proportion of ~ 6.0% (**Figures 2K, L**).

To assess the heterogeneity among individuals, we analyzed CMV-specific responses in the peripheral blood of additional donors. Participant #2 had NLV^+^ tetramer cells that constituted 0.6% of total CD3^+^ T cells (**Figure 2M**) and 1.4% of total CD8^+^ T cells (**Figure 2N**). In this participant, NLV-specific CD8^+^ T cells were CX3CR1^+^ and GPR56^+^, with much less CD57 expression (**Figure 2O**). There was also a higher proportion of TEM cells among the gated NLV tetramer^+^ cells (**Figure 2P**). NLV tetramer-specific cells in participant #2 did not form a tight cluster as seen with participant #1 (**Figure 2Q**), and 1.9% of CGC^+^ CD8^+^ T cells were NLV-tetramer specific (**Figure 2R**). Two additional HLA-A*02:01 PWH were also evaluated: participant #3 with 2.1% NLV tetramer^+^ cells as a proportion of total CD3^+^ T cells (**Supplemental Figures 3A-F**) and participant #4 with 0.8% NLV tetramer^+^ cells as a proportion of total CD3^+^ T cells (**Supplemental Figures 3G-L**). In summary, despite heterogeneity in immune cell markers, NLV-tetramer^+^ CD8^+^ T cells are largely present within the CGC^+^ cluster.

### CMV-specific CD4^+^ T cells co-express CX3CR1 and GPR56 with variable CD57

**Figure 3:**
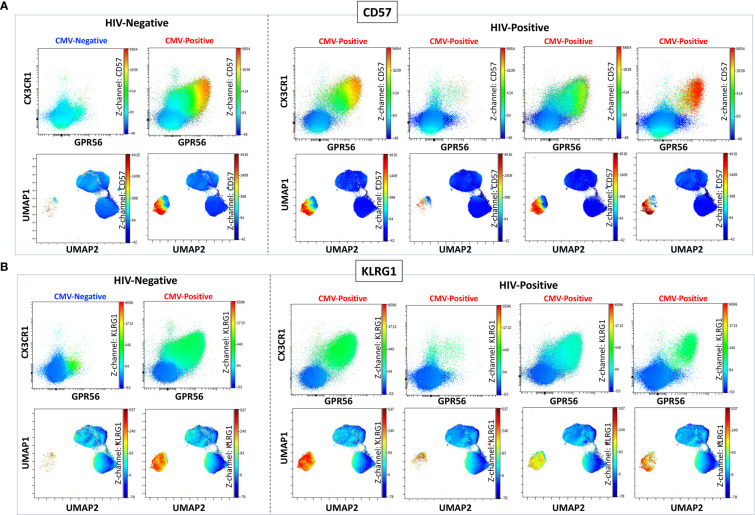
CGC^+^ CD4^+^ T cells express CX3CR1 and GPR56 with variable expression of CD57. The two-dimensional flow cytometry panel shows the co-expression of CX3CR1 and GPR56 and highlights CD57 expression on these cells compared to non-CGC^+^ T cells. UMAP shows CGC^+^ CD4^+^ cluster and variable expression of CD57 **(A)**. KLRG1 expression on CGC^+^ CD4^+^ T cells is demonstrated by two-dimensional plots and UMAP **(B)**. Participants in this analysis included two HIV-negative persons with and without CMV, and four CMV-positive PWH.

**Figure 4:**
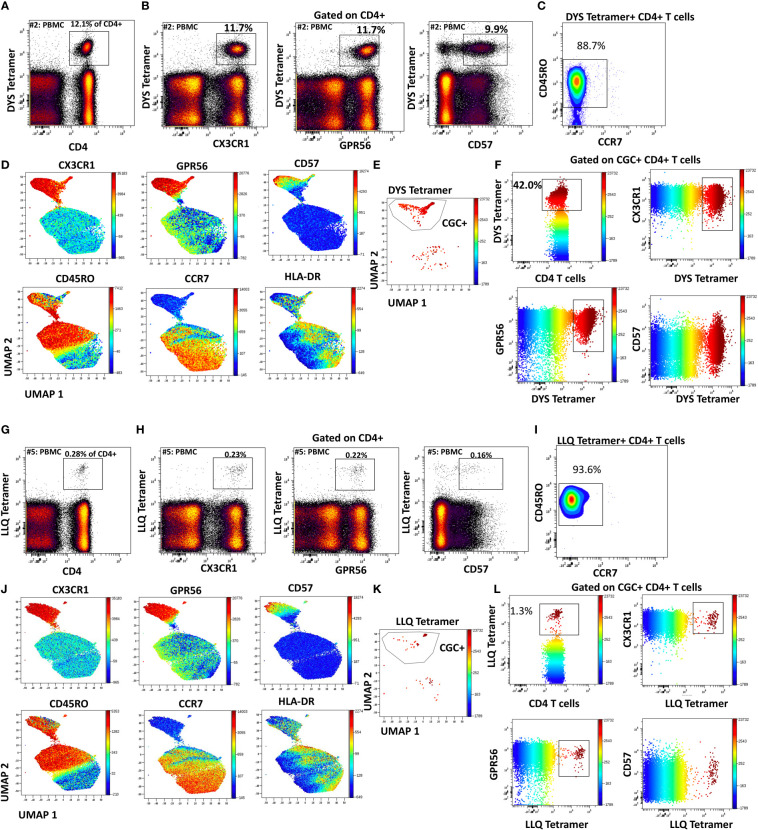
CMV-specific CD4^+^ T cells are predominantly CGC^+^. Phenotypic expression of CX3CR1, GPR56, and CD57 by CMV-specific CD4^+^ T cells that recognize two different epitopes (DYS and LLQ). Peripheral blood DYS-specific CD4 T cells as a proportion of CD3+ T cells **(A)**. Two-dimensional plots show the co-expression of CX3CR1, GPR56, and CD57 by DYS-specific CD4 T cells **(B)**. Memory cell phenotypes were classified as TEM (CD45RO^+^ CCR7^-^) and TEMRA (CD45RO- CCR7-) **(C)**. UMAP of CD4^+^ T cells showing the CD4^+^ T cells with DYS tetramers among the CGC cells. Each panel shows the distribution of CX3CR1, GPR56, CD57, CD45RO, CCR7, and HLA-DR expression on the clusters **(D-F)**. A second participant with LLQ-specific CD4^+^ T cells is shown **(G-L)**.

Like CGC^+^ CD8^+^ T cells, we also characterized markers expressed on CGC^+^ CD4^+^ T cells. The two-dimensional plots show CX3CR1 and GPR56 expression on the y and x-axis and highlight CD57 expression on the z-channel (**Figure 3A**). The CMV-negative donor had very few CGC^+^ CD4^+^ T cells, unlike CGC^+^ CD8^+^ T cells. The UMAP shows the separation of the CGC^+^ CD4^+^ T cell cluster from the rest of the CD4^+^ T cells. In addition, CGC^+^ CD4^+^ T cells also express KLRG1, which may be less variable than CD57 (**Figure 3B**). With additional markers, we can define CGC+ CD4+ T cells as TEM (CD45RO^+^ CCR7^-^) and TEMRA (CD45RO^-^ CCR7^-^) cells that are CD28^+/-^, CD27^-^, PD1^+/-^ and CD38^+/-^(**Supplemental Figure 5**). We used MHC Class II tetramers to identify CD4^+^ T cells recognizing two immunodominant CMV epitopes: DYS and LLQTGIHVRVSQPSL (LLQ, HLA-DQ06:02, pp65 protein) as previously published (10, 13). Participants with HLA-DR7 and HLA-DQ6 were selected (**Supplemental Table 2**). 12.1% of the total CD4^+^ T cells in participant #2 were DYS tetramer^+^ (**Figure 4A**). Two-dimensional flow cytometry plots show that DYS-tetramer^+^ CD4^+^ T cells also express CX3CR1 and GPR56 with variable CD57 (**Figure 4B**). 88.7% of the DYS-tetramer^+^ CD4^+^ T cells were TEM, and the rest were TEMRA (**Figure 4C**). Visualization using the UMAP technique showed that the majority of the DYS tetramer^+^ cells were within the CGC^+^ CD4^+^ cluster (**Figures 4D, E**). Analysis of the DYS tetramer on the CGC^+^ CD4^+^ cluster showed that 42.0% of CGC^+^ CD4^+^ T cells in participant #1 had TCRs that recognized the DYS epitope (**Figure 4F**). In participant #5 (**Supplemental Table 2**, HLA-DQ6+) LLQ-specific CD4^+^ T cells comprised 0.28% of CD4^+^ T cells (**Figure 4G**). LLQ tetramer^+^ T cells co-expressed CX3CR1, and GPR56 with variable CD57 expression (**Figure 4H**). 93.6% tetramer^+^ cells fell within the CGC^+^ cluster (**Figures 4I-K**). 1.3% of CGC^+^CD4^+^ T cells in participant #5 were specific for the LLQ epitope (**Figure 4L**). In summary, the majority of the CD4^+^ T cells with TCRs that recognize two different immunodominant CMV epitopes are CGC^+^. CMV-specific CD4^+^ T cells in PWH are significantly inflated compared to matched HIV-negative controls (13). These data suggest that in PWH without evidence of acute CMV infection at the time of the study, a large proportion of CGC^+^ T cells may be CMV-specific.

### Detection of low-frequency CMV-specific CD4^+^ T cells among expanded CGC^+^ T cells

**Figure 5:**
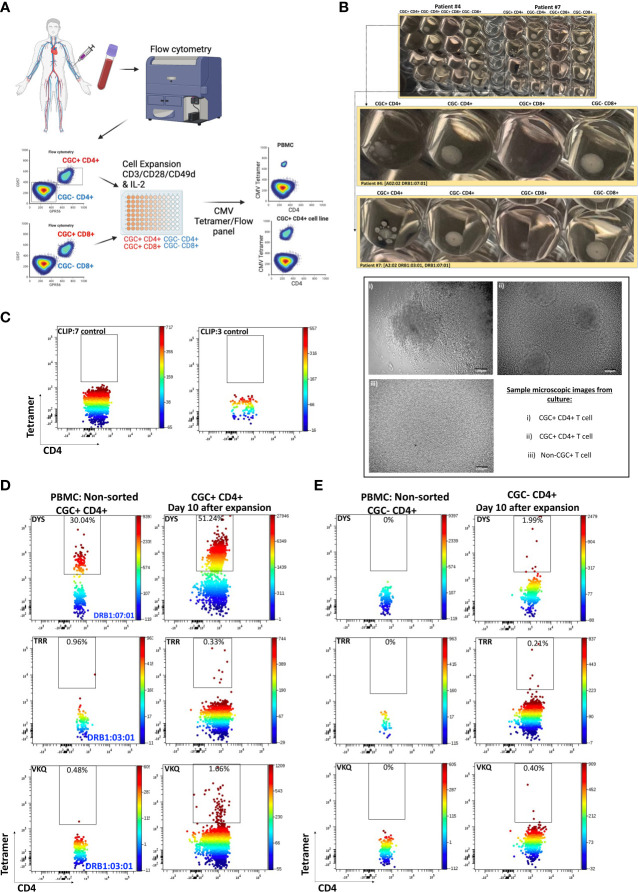
CMV-specific T cells among expanded CGC^+^ CD4^+^ and non-CGC^+^ CD4^+^ T cells. Schematic showing workflow for sorting and expansion of CGC^+^ and non-CGC^+^ cells **(A)**. Cell expansion cultures from two participants (**Supplemental Table 2**, participants #4 & #7) on day 10 of expansion. The top figure shows duplicate wells, with the middle panel showing an enlarged image to highlight the satellite cultures in CGC^+^ CD4^+^ T cells by direct observation and by light microscopy **(B)**. Tetramer staining controls on expanded cells (DRB1:07:01) and DRB1:03:01) with CLIP peptide **(C)**. Two-dimensional flow cytometry plots showing CMV-specific T cells using tetramers against three different CMV MHC class II epitopes (DR7:DYS) and (DR3:TRR, DR3:VKQ) in non-sorted PBMCs and expanded CGC^+^ CD4^+^ T cells **(D)**. Similar analysis on non-CGC^+^ CD4^+^ T cells gated from PBMCs and on expanded non-CGC^+^ CD4+ T cell line **(E)**.

To characterize CMV-specific T cells with TCRs to less immunodominant epitopes with a lower frequency of tetramer^+^ T cells in PBMCs, we flow-sorted and expanded CGC^+^ and non-CGC^+^ T cells as depicted in the schematic (**Figure 5A**). We made attempts to expand CGC^+^ CD8^+^ T cells but could not analyze this population due to a high proportion of cell death. (Photos of expanded cell subsets on day 10 of culture are shown in **Figure 5B**). The morphology of the expanded CGC^+^ CD4^+^ T cells in some participants was distinct from the non-CGC^+^CD4^+^ and CD8^+^ T cells, with satellite clusters that we speculate may represent clonal expansion. Control MHC class II tetramers (HLA-DR3 and HLA-DQ6) with the CLIP peptides were also used to stain CGC^+^ CD4^+^ T cells from expanded cell lines (**Figure 5C**). We used the CMV tetramers to identify CD4^+^ T cells with TCRs to less dominant epitopes (TRRGRVKIDEVSRMF (TRR, HLA-DRB1*03:01, IE2 protein) and VKQIKVRVDMVRHRI (VKQ, HLA-DRB1*03:01, IE1 protein)). We measured tetramer^+^ CD4^+^ T cells (DYS, TRR, and VKQ) after a 10-day expansion of sorted CGC^+^ CD4^+^ T cells and compared them to unsorted PBMCs (**Figure 5D**). For some of the less dominant epitopes, we had improved detection after expansion in culture (TRR and VKQ). Sorted non-CGC^+^ CD4^+^ T cells expanded in culture for 10 days also had some CMV-specific CD4^+^ T cells by tetramer analysis (**Figure 5E**). Expanded CGC^+^ CD4^+^ cells maintained CX3CR1, GPR56, and CD57 expression on the tetramer^+^ cells (**Supplemental Figures 5A, C, E**). The tetramer^+^ CD4^+^ T cells that we detected in the expanded T cells from the non-CGC^+^ CD4^+^ sort also expressed CX3CR1 and GPR56 compared to the other cells in the same pool. This may suggest that some CGC^+^ T cells were among the non-CGC^+^ T cells obtained by sorting before expansion, or that CGC^+^ CD4^+^ T cells may be derived from non-CGC^+^ T cells (**Supplemental Figures 5B, D, F**). In general, CGC^+^ CD4^+^ T cells maintained higher levels of CX3CR1, GPR56, and CD57 in culture (**Supplemental Figures 5G, H**). Notably, there was no significant difference in the mean fluorescence intensity of the CX3CR1 and GPR56 between the tetramer^+^ cells in the CGC^+^ CD4^+^ population versus tetramer^+^ cells in the non-CGC^+^ CD4^+^ expanded T cells, while CD57 expression trended towards being higher in the expanded CGC^+^ CD4^+^ T cells (**Supplemental Figure 5I**). Taken together, CGC^+^ CD4^+^ T cells as we have defined them can proliferate in culture after CD3/CD28 stimulation with IL-2 supplementation. This is different from previous studies that failed to show the proliferation of CD57^+^ CD4^+^ T cells after stimulation with PHA (42) or HIV antigens (43). In our studies, the CGC^+^ CD4^+^ T cell cluster appears to be driven by CX3CR1 and GPR56, which includes CD4^+^ T cells with variable CD57^+^ T cell expression that may undergo several rounds of replication. In summary, CMV-tetramer^+^ CD4^+^ T cells in PWH are mainly CX3CR1^+^ GPR56^+^ with variable expression of CD57, while similar cells present in the non-CGC^+^ cells have low expression of CD57.

### CGC^+^ CD4^+^ T cells have a large proportion of clonal TCRs and are largely CMV-specific

**Figure 6:**
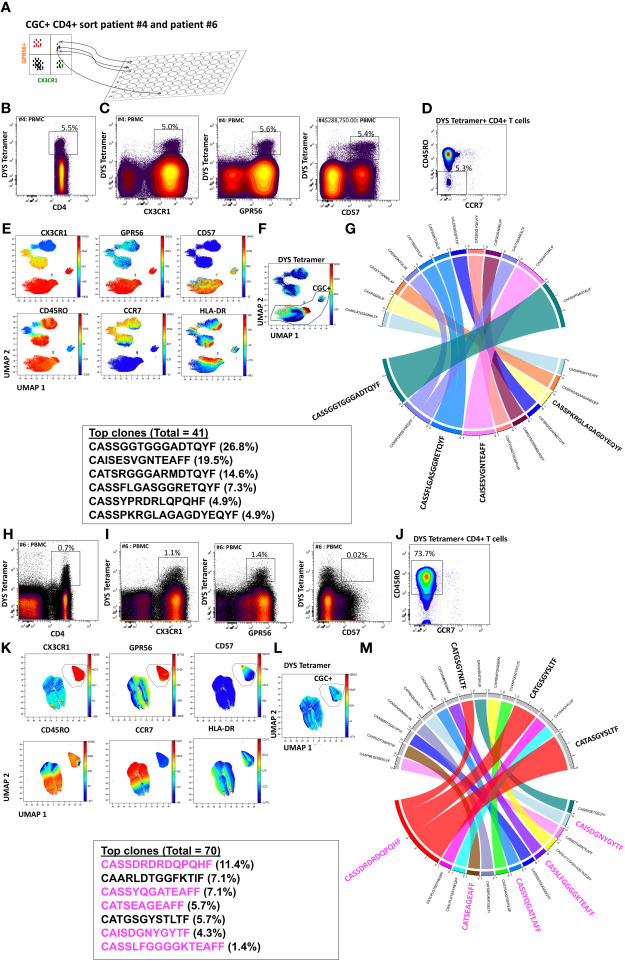
CGC^+^ CD4^+^ T cells express clonal TCRs and are largely CMV-specific. Single CGC^+^ CD4^+^ T cells were sorted into 96 well plates as shown **(A)**. Two-dimensional plots from participant #4 show DYS tetramer-specific T cells as a proportion of CD4^+^ T cells **(B)**. CX3CR1, GPR56, and CD57 expression on DYS tetramer^+^ cells **(C)** and memory distribution of the tetramer^+^ cells are shown **(D)**. UMAPs show the distribution of markers **(E, F)**. A total of 40 paired TCRs had identifiable sequences. The Circos plot shows paired αβ TCR CDR3 sequences, and TRβV and paired TCRJ genes are shown **(G)**. A similar analysis was done with participant #6 **(H-M)**. CDR3 sequences in magenta have previously been shown to be DYS-specific, by tetramer staining.

While we observed that CGC^+^ CD4^+^ T cells can recognize several CMV epitopes, it remains unclear the extent to which the CGC^+^ T cell cluster of cells is CMV-specific as a whole. To understand whether starting with CGC^+^ CD4^+^ T cells can help define CMV-specific T cells agnostic to HLA typing, we sorted single CGC^+^ CD4^+^ T cells into 96-well plates from two participants (#4 and #6, both HLA-DR7) as shown (**Figure 6A**). Two-dimensional plots show that 5.5% of CD4^+^ T cells in participant #4 are DYS tetramer^+^ (**Figures 6B, C**) and largely TEM cells (**Figure 6D**). UMAPs show DYS tetramer^+^ T cells within the CGC^+^ T cell cluster (**Figures 6E, F**). Paired αβ TCR sequences from the sorted CGC^+^ CD4^+^ T cells are shown in Circos plots (**Figure 6G**). Clonal TCRs that did not have paired αβ pairs are not shown on the Circos plots. Out of a total of 41 TCRs with paired αβ pairs, 26.8% had the CDR3 (CASSGGTGGGADTQYF). Other clonal TCRs are shown. Participant #6 was selected because of known DYS-specific CD4^+^ TCRs identified by bulk sequencing apriori (13). We also sorted CGC^+^CD4^+^ T cells from participant #6 (**Figures 6H-M**). Out of a total of 70 TCR sequences with matched αβ TCR pairs, 5 of the top 10 clones among the CGC^+^ CD4^+^ T cells (n=21) had been previously identified as CMV-DYS specific by sequencing TCRs from DYS tetramer^+^ CD4^+^ T cells (13). This suggests CGC^+^ CD4^+^ T cells in PWH co-infected with CMV may be largely CMV-specific.

The frequency of peripheral blood CGC^+^ CD4^+^ T cells from participants in the full cohort of PWH was positively correlated with plasma anti-CMV IgG titers (ρ=0.21, *p*=0.03), while CGC^+^ CD8^+^ T cells were not significant (ρ=0.18, *p*=0.07) (**Supplemental Figures 6A, B**). Finally, CGC^+^ CD4^+^ T cells and CGC^+^ CD8^+^ T cells were strongly correlated with each other (**Supplemental Figure 6C**).

### CGC^+^ CD4^+^ T cells are higher in PWH with cardiometabolic disease

**Figure 7:**
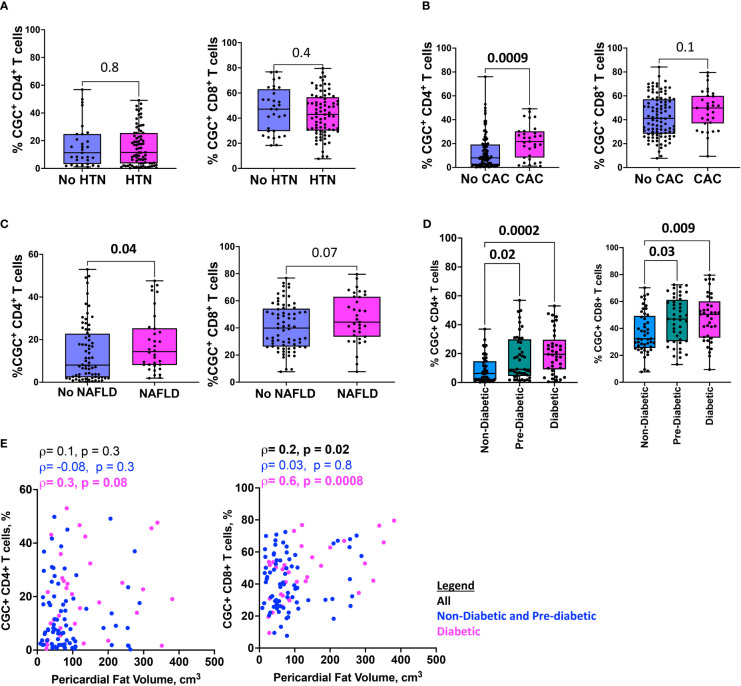
HIgher CGC^+^ CD4^+^ T cells are associated with cardiometabolic disease. Box plots showing CGC^+^ CD4^+^ and CGC^+^ CD8^+^ T cells in the presence or absence of hypertension (HTN) **(A)**, coronary arterial calcium (CAC) **(B)**, non-alcoholic fatty liver disease (NAFLD) **(C)**, and diabetes **(D)**. Correlation plots show the relationship between CGC^+^ T cells and pericardial fat volume **(E)**. Statistical analysis by Mann Whitney, Kruskal Wallis, and Spearman’s rank correlation tests.

To investigate the extent to which CGC^+^ CD4^+^ and CGC^+^ CD8^+^ T cells differ in cardiometabolic disease conditions (diabetes, pre-diabetes, hypertension, coronary arterial calcium, nonalcoholic fatty liver disease, and pericardial fat volume), we measured the frequencies of CGC^+^ T cells in PBMCs as a proportion of total CD4^+^ and CD8^+^ T cells. We found similar proportions of CGC^+^ CD4^+^ and CGC^+^ CD8^+^ T cells in participants with and without hypertension (**Figure 7A**), while CGC^+^ CD4^+^ T cells were higher in participants with coronary arterial calcium (CAC) on CT imaging (p=0.0009), (**Figure 7B**), and non-alcoholic fatty liver disease (NAFLD, defined as absolute liver attenuation less than 58 Hounsfield units [HU] on CT imaging [p=0.04; **Figure 7C**]). Both CGC^+^ CD4^+^ and CGC^+^CD8^+^ T cells were significantly higher among participants with prediabetes and diabetes as compared to non-diabetics (**Figure 7D**). Notably, the frequency of CGC^+^ CD8^+^ T cells was positively correlated with pericardial fat volume as measured by CT imaging (p=0.02), which was mainly driven by diabetic participants (p=0.0008) (**Figure 7E**). A similar relationship was not observed for CGC^+^ CD4^+^ T cells and pericardial fat volume (p=0.3). Collectively, these results indicate that there are more circulating CGC^+^ CD4^+^ and CGC^+^ CD8^+^ T cells in persons with HIV with subclinical atherosclerosis, NAFLD, pre-diabetes, and diabetes.

### Circulating CGC^+^ CD4^+^ and CGC^+^ CD8^+^ T cells are associated with fasting blood glucose and hemoglobin A1C

**Figure 8:**
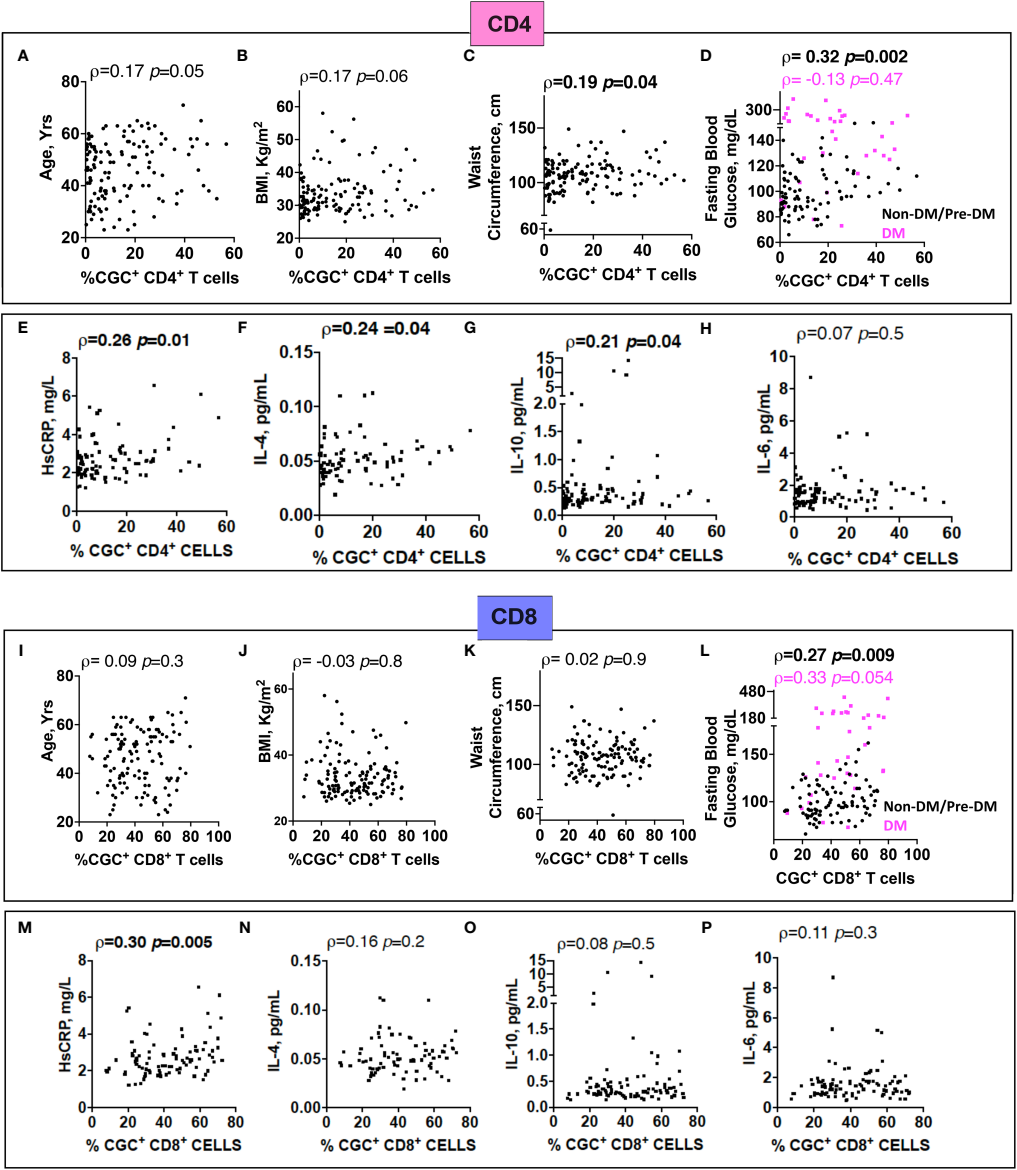
The proportion of circulating CGC^+^ T cells in peripheral blood is correlated with fasting blood glucose. Spearman’s rank correlation analysis shows relationships between CGC^+^ CD4^+^ T cells and age **(A)**, body mass index (BMI) **(B)**, waist circumference **(C)**, and fasting blood glucose stratified by non-diabetic and prediabetic (black) and diabetic (magenta) **(D)**. Correlation plots showing relationships between % CGC^+^ CD4^+^ T cells and inflammatory markers including high sensitivity C-reactive protein (HsCRP) **(E)**, IL-4 **(F)**, IL-10 **(G)**, and IL-6 **(H)**. Similar analyses were performed with CGC^+^ CD8^+^ T cells **(I-P)**. Statistical analysis by Spearman’s rank test.

**Figure 9:**
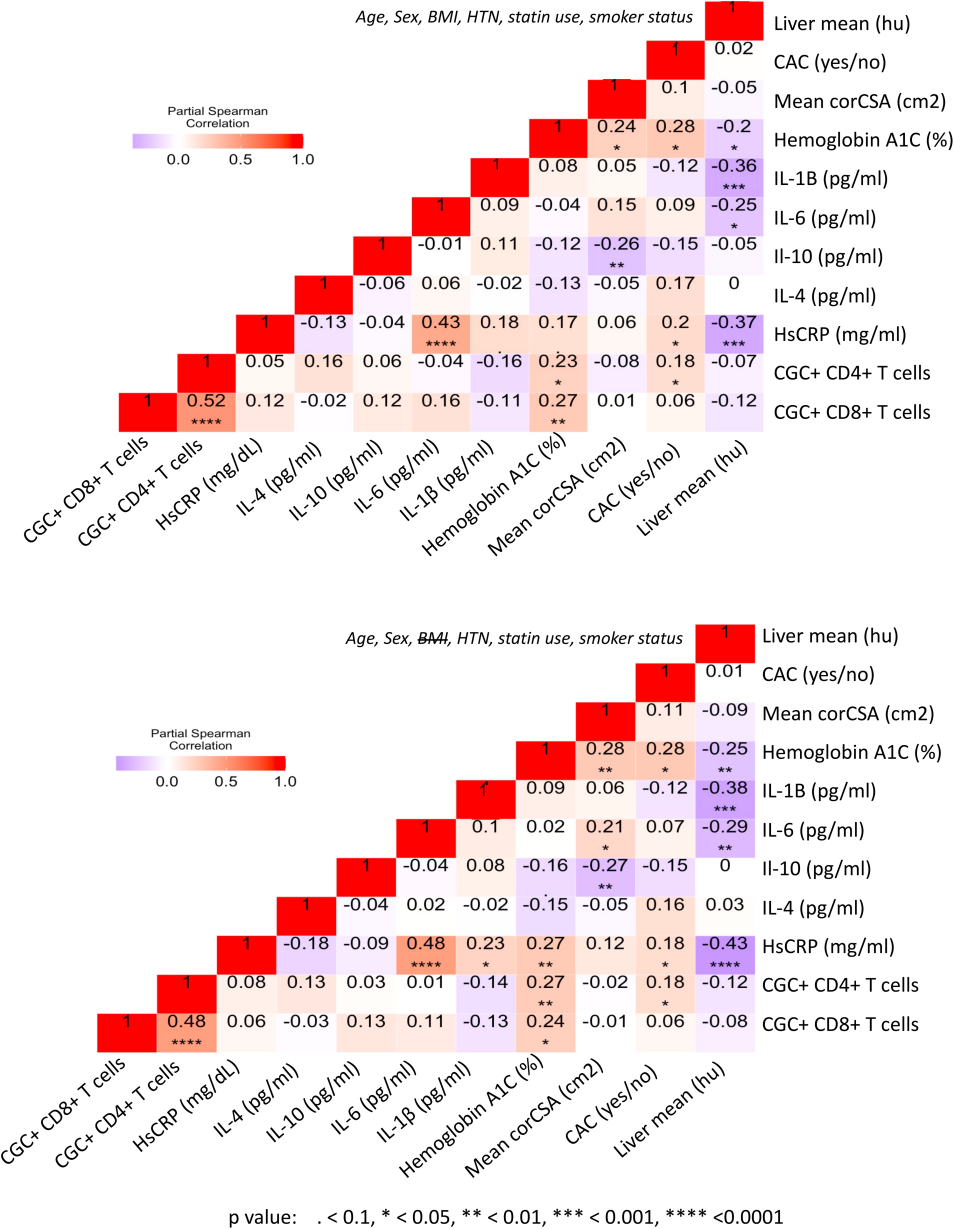
CGC^+^ T cells are associated with higher hemoglobin A1C levels in adjusted analyses. Partial Spearman’s rank correlation analysis adjusted for age, sex, HTN, BMI, statin use, and smoking status (top), and after removal of BMI from the model (bottom).

Prior murine studies showed the adoptive transfer of peripheral senescent CD8^+^ T cells (CD8^+^ CD44^+^ CD153^+^) from spleens of mice on a high-fat chow diet to mice on a normal chow diet was followed by insulin resistance in the recipient animals, suggesting a role for circulating T cells in the development of metabolic dysregulation (44). This concept is further supported by epidemiologic analyses in PWH showing an association between a higher circulating CD4^+^ TEMRA cell frequency and the subsequent development of diabetes mellitus (45). In light of these findings, we assessed the relationship between peripheral CGC^+^ CD4^+^ T cells (as a proportion of total CD4^+^ T cells) and CGC^+^ CD8^+^ T cells (as a proportion of total CD8^+^ T cells) with several cardiometabolic disease risk factors. CGC^+^ CD4^+^ T cells were correlated with waist circumference, and there was a trend towards a correlation with age and BMI (**Figures 8A–C**). These cells were most strongly correlated with fasting blood glucose in non-diabetic and pre-diabetic individuals (*p*=0.002) (**Figure 8D**). The correlation with fasting blood glucose was stratified by the metabolic group because of the effect of diabetes medications on glucose levels (all diabetic participants were receiving anti-diabetes medications and had a fasting blood glucose range of 73-416 mg/dL). We did not observe a significant relationship between CGC^+^ CD4^+^ T cells and fasting blood glucose in diabetic PWH (=-0.13, *p*=0.47). T cells can mediate inflammatory and anti-inflammatory effects through plasma cytokines. Therefore, we also assessed whether CGC^+^ T cells were related to inflammatory markers and anti-inflammatory cytokines important in cardiometabolic disease (35). The frequency of CGC^+^ CD4^+^ T cells correlated with hsCRP (p=0.01), IL-4 (p=0.04), IL-10 (p=0.04) but not IL-6 (p=0.5) (**Figures 8E-H**). On the other hand, CGC^+^ CD8^+^ T cells were not correlated with age, BMI, or waist circumference, and had a modest correlation with fasting blood glucose in non-diabetic/pre-diabetic PWH (r=0.27, p=0.009), and a trend toward significance in diabetic PWH (r=0.33, p=0.05) (**Figures 8I-L**) CGC^+^ CD8^+^ T cells were also correlated with hsCRP (ρ=0.30, *p*=0.005) (**Figure 8M**) but not IL-4, IL-10, or IL-6 (**Figures 8N-P**). Both CGC^+^ CD4^+^ T cells (ρ=0.23, p=0.01) and CGC^+^ CD8^+^ T cells (ρ=0.27, p=0.003) were correlated with hemoglobin A1C in a partial Spearman’s correlation analysis adjusted for age, sex, body mass index (BMI), hypertension, statin use, and smoking status (**Figure 9**, top). CGC^+^ CD4^+^ T cells had a stronger association with hemoglobin A1C when BMI was removed from the model (ρ=0.27, p=0.004), while the relationship between CGC^+^ CD8^+^ T cells and hemoglobin A1C attenuated (ρ=0.24, p=0.01) (**Figure 9**, bottom).

### CGC^+^ CD4^+^ and CGC^+^ CD8^+^ T cells are correlated with circulating concentrations of starch/sucrose metabolites and branch-chain amino acids

**Figure 10:**
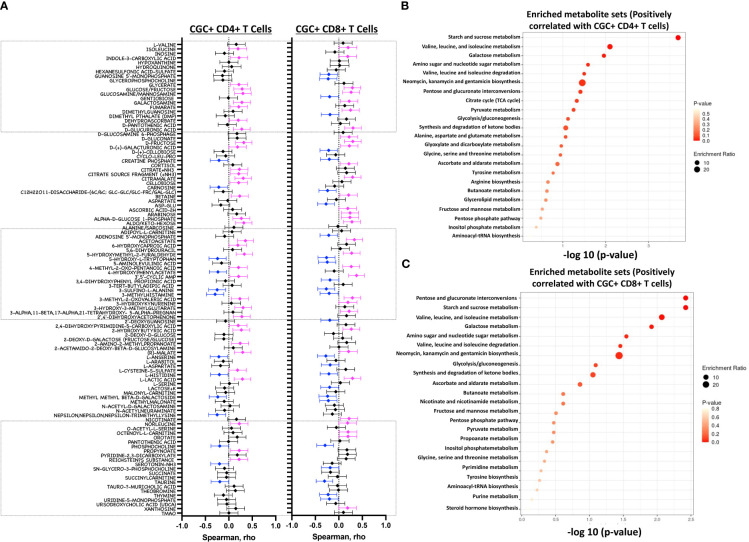
Starch and sucrose metabolism pathways are enriched among plasma metabolites positively correlated with CGC^+^ T cells. Forest plots showing Spearman’s rank correlation coefficients between plasma metabolites and the proportion of CGC^+^ CD4^+^ T cells over total CD4^+^ T cells (left panel) and % CGC^+^ CD8^+^ T cells over total CD8^+^ T cells (right panel) **(A)**. The top twenty-five metabolite sets in the enrichment analysis (number of metabolites/expected metabolites per set) were determined based on metabolites that were positively correlated with % CGC^+^ CD4^+^ T cells **(B)** and % CGC^+^ CD8^+^ T cells **(C)**. Statistical analysis by Spearman’s rank correlation. Color code: Blue, negative correlation p<0.05; Magenta, positive correlation p<0.05; black, non-significant correlation. Over Representation Analysis (ORA) of plasma metabolites that were associated with CGC^+^ T cells was performed using MetaboAnalyst 5.0 with the hypergeometric test. One-tailed adjusted p values are provided after correcting for multiple comparisons.

Both fasting blood glucose and HbA1c can be influenced by multiple factors, including the time of day, hydration status, medications, red blood cell survival, genetics, and vitamin deficiencies, among others. Plasma metabolites offer an additional profile of metabolic status, and changes in metabolites are frequently present before the development of overt disease (46). Therefore, we next assessed whether the observed association between CGC^+^ T cells and blood glucose measurements was also present for other plasma metabolites. We found that frequencies of CGC^+^ CD4^+^ T cells and CGC^+^ CD8^+^ T cells were positively correlated with plasma levels of branch chain amino acids (isoleucine and norleucine), carbohydrate metabolites (glucose/fructose, glucosamine, galactosamine, and fumarate), acetoacetate, and 2-beta-hydroxybutyric acid among others (**Figure 10A**). On the other hand, phosphocholine, L-Anserine, 3-Methylhistamine, Sulfino-L-Alanine, and Hydroxy-L-Tryptophan were inversely correlated with the frequencies of CGC^+^ CD4^+^ and CGC^+^ CD8^+^ T cells. Most of these correlations were not present for total memory CD4^+^ or CD8^+^ T cells (**Supplemental Figures 7A, B**). The frequency of CGC^+^ CD4^+^ cells positively correlated with metabolites that enriched for the starch and sucrose metabolism pathways (FDR 0.02) (**Figure 10B**), and negatively with other metabolites from the taurine and hypotaurine metabolism pathways (FDR 0.09, data not shown). Metabolites that were positively correlated with CGC^+^ CD8^+^ cells enriched for the pentose and glucuronate conversions, starch, and sucrose metabolism pathways but were not statistically significant (FDR 0.16) (**Figure 10C**). Similarly, metabolites that were negatively correlated with CGC^+^ CD8^+^ enriched for taurine and hypotaurine metabolism pathways but they were not significant (FDR 0.33, data not shown). Taken together, these results demonstrate that CGC^+^ T cells are related to starch and sucrose metabolites in plasma, as well as branched-chain amino acids (BCAA) which have been linked with the development of diabetes and cardiovascular disease (46–48).

### CGC^+^ CD4^+^ and CGC^+^ CD8^+^ T cells are predominantly mitochondrial-dependent

**Figure 11:**
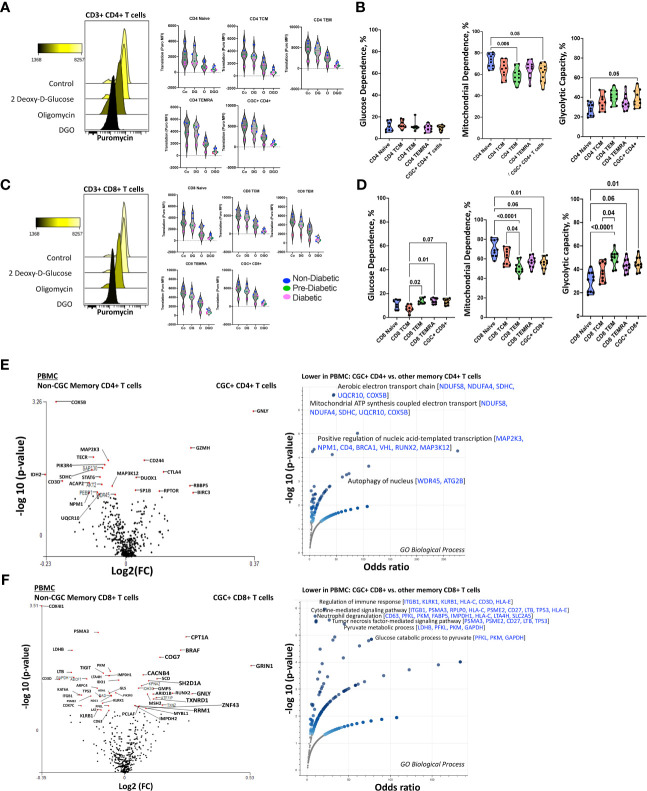
Unstimulated CGC^+^ CD4^+^ and CGC^+^ CD8^+^ T cells are dependent on oxidative phosphorylation. Overlapping histograms showing puromycin geometric mean fluorescence in CD4^+^ T cells after treatment with 2 deoxy-d-glucose (2DG), oligomycin (o), 2DG, and oligomycin (DGO), and media (Co). Adjacent violin plots show the geometric mean fluorescence (MFI) of puromycin in different memory subsets for the different experimental conditions (Co, DG, O, and DGO groups), and participants by color-coded by diabetes status **(A)**. Truncated violin plots showing the glucose dependence, mitochondrial dependence, and glycolytic capacity in all memory subsets including CGC^+^ CD4^+^ T cells **(B)**. Similar analyses were done with CD8^+^ T cells showing differences in puromycin uptake in all memory subsets **(C)** and differences in glucose dependence, mitochondrial dependence, and glycolytic capacity **(D)**. Volcano plots show differential gene expression of metabolic genes between CGC^+^ CD4^+^ and non-CGC CD4^+^ T cells **(E)**, and CGC^+^ CD8^+^ and non-CGC^+^ CD8^+^ T cells **(F)**. Statistical analysis **(B, D)** was performed using the Mann-Whitney U test and differential gene expression by the Kruskal-Wallis test. GO Biological Processes (GO) enrichment was performed using Enrichr with multiple comparisons correction (**Supplemental Tables S5, S7**) (38–40).

CD4^+^ TEMRA cells are senescent and bioenergetically flexible compared to CD8^+^ TEMRA cells, partly due to their ability to effectively engage both glucose metabolism and oxidative phosphorylation (49). One possible explanation is that CD8^+^ TEMRA cells may have dysfunctional mitochondria (49). Given the observed relationship between the frequency of CGC^+^ T cells with starch and sucrose metabolites, we studied their metabolic profile as a way to understand their functional capacity in PWH. We analyzed the mitochondrial and glucose dependence of T cells using SCENITH, Single Cell ENergetIc metabolism by profiIing Translation in inHibition. Puromycin uptake by CGC^+^ CD4^+^ and CGC^+^ CD8^+^ T cells after incubation with inhibitors was measured by geometric mean fluorescence (**Figures 11A, C**). In general, naïve cells (CD4^+^ and CD8^+^) had lower levels of puromycin uptake compared to T cell memory subsets (**Figures 11A, C**). We found that unstimulated CD4^+^ memory (TCM, TEM, TEMRA, and CGC^+^) T cell subsets were predominantly mitochondrial-dependent (**Figure 11B**). CD4^+^ naïve T cells had higher mitochondrial dependence compared to CD4^+^ TEM (*p*=0.006). Although not significant, CGC^+^ CD4^+^ T cells had lower mitochondrial dependence and higher glycolytic capacity when compared to CD4^+^ naïve T cells (*p*=0.05) (**Figure 11B**). CD8^+^ T cell memory subsets also had higher mitochondrial dependence. However, CD8^+^ TEM (p=0.02) and TEMRA (p=0.01) cells showed significantly higher glycolytic dependence than central memory CD8^+^ T cells (TCM) (**Figure 11D**). Unstimulated CGC^+^ CD8^+^ T cells also had higher glucose dependence than CD8^+^ TCM but this was not significant (p=0.07) (**Figure 11D**). CGC^+^ CD4^+^ T cells that we expanded in culture using CD3/CD28/CD49d did not utilize glycolysis as effectively as non-CGC^+^ T cells (**Supplemental Figures 8A, B**). Notably, expanded CGC^+^ CD8^+^ T cells utilized glycolysis more than CGC^+^ CD4^+^ T cells (**Supplemental Figure 8C**). Taken together, since T cells are known to be bioenergetically flexible and can undergo metabolic reprogramming to utilize the more abundant nutrition sources in the local environment, it is possible that CGC^+^ T cells can meet their bioenergetic demands using abundant nutrient sources other than glucose.

Transcriptionally, unstimulated CGC^+^ CD4^+^ T cells were enriched for cytotoxic RNA transcripts (*GNLY, CD244, GZMH, CTLA4*) and were deficient in transcripts that form the aerobic/mitochondrial electron transport chain [*SDHC (complex II), UQCR10 (complex III), COX5B (complex IV)*] when compared to non-CGC^+^ CD4^+^ memory T cells (**Figure 11E** and **Supplemental Tables 4, 5**). CGC^+^ CD8^+^ T cells, on the other hand, displayed higher *CPT1A* (fatty acid β oxidation), *GRIN1* (glutamate receptor), and lower *COX4I1* and *COX7C* transcripts than non-CGC^+^ CD8^+^ T cells (**Figure 11F** and **Supplemental Tables 6, 7**). Some studies have suggested that long-chain fatty acid oxidation modulated by CPT1A is important for CD8^+^ T cell memory development, while knockout studies in mice suggest that CPT1A is dispensable (50).

We performed a direct comparison of CGC^+^ CD4^+^ and CGC^+^ CD8^+^ T cell transcriptomes to try and explain why CGC^+^ CD4^+^ T cells appeared to have a stronger relationship with cardiometabolic disease conditions and risk factors. We found that CGC^+^ CD8^+^ T cells were enriched for genes involved in mitochondrial ATP synthesis coupled electron transport [*SDHC, UQCR10, COX5B*] and fatty-acyl-CoA biosynthetic process [*TECR*] (**Supplemental Figures 8A-C** and **Supplemental Table 6**). Notably, three genes, *SDHC, UQCR10,* and *COX5B*, were consistently lower in CGC^+^ CD4^+^ T cells as compared to other memory CD4^+^ T cells and CGC^+^ CD8^+^ T cells. While CGC^+^ CD4^+^ T cells had higher expression of several genes including *SLC16A1*, *BCL2, BIRC3, ICOS, CTLA4,* and *IDO1* which enriched several pathways including the pyruvate metabolic process. Taken together, the RNA transcriptomes of both CGC^+^ CD4^+^ and CGC^+^ CD8^+^ T cells show lower expression of transcripts that encode for mitochondrial complexes than non-CGC^+^ T cells. However, a direct comparison between CGC^+^ CD8^+^ and CGC^+^ CD4^+^ T cells suggests differences in bioenergetic sustenance. Notably, CGC^+^ T cells are largely TEM and TEMRA, compared to other memory cells which would include cells that are TCM, TEM, and TEMRA. Differences that may be driven by TCM in the non-CGC^+^ memory T cells have not been accounted for in this analysis.

### CGC^+^ T cells express higher levels of carnitine palmitoyl transferase than other memory T cells

**Figure 12:**
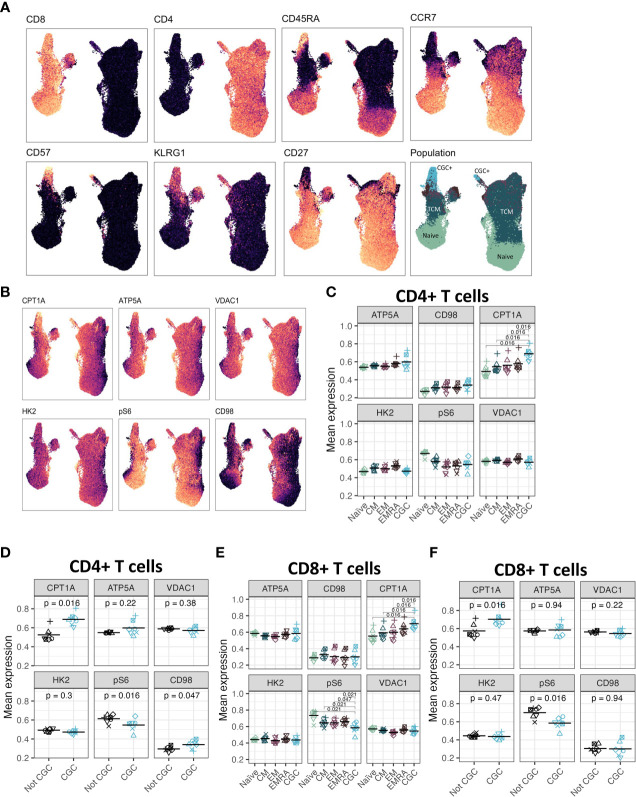
CGC^+^ CD4^+^ T cells have higher expression of carnitine palmitoyl transferase (CPT1A), a rate-limiting enzyme of fatty acid oxidation. UMAPs showing markers used to define the CGC^+^ T cells (CD28^-^ KLRG1^+^ CD27^-^) **(A)**. CPT1A, ATP5A, VDAC1, HK2, pS6, and CD98 expression were measured by mass cytometry **(B)**. Dot plots show the expression of each of the metabolic markers between CD4^+^ naive, central memory (CM), effector memory (EM), TEMRA (EMRA), and CGC T cells **(C)**, as well as a comparison of CGC^+^ CD4^+^ T cells and non-CGC^+^ T cells [every other CD4+ T cell] **(D)**. A similar analysis is shown with CD8^+^ T cell subsets **(E, F)**. Statistical analysis **(C, E)** was performed using pairwise comparisons between the CGC subset and each of the other four populations for each marker, followed by local FDR for multiple hypothesis correction within each marker for the four population comparisons. We only show comparisons with q value<0.05. Statistical analysis of **(D, F)** was by paired Wilcoxon signed rank test, with no multiple hypothesis testing.

Although CGC^+^ CD4^+^ and CGC^+^ CD8^+^ T cells were modestly correlated with fasting blood glucose, we did not find significant differences in glucose dependence compared to other memory T cells. Transcriptional analysis suggested that CGC^+^ CD8^+^ T cells may rely on long-chain fatty acid oxidation mediated by CPT1A. A previous study showed that PD-1 ligation of T cells induced CPT1A expression leading to an increased rate of fatty acid β oxidation (FAO) while limiting glycolysis (51). Some CGC^+^ CD4^+^ T cells and CGC^+^ CD8^+^ T cells express PD-1 (**Supplemental Figures 2, 4**), and our differential gene expression analysis suggests that CGC^+^ CD8^+^ T cells may use FAO as an energy source. We leveraged a separate cohort of HIV-negative donors that had undergone single-cell metabolic profiling of CD4^+^ and CD8^+^ T cells by mass cytometry (29). In this prior cohort, study, we defined CGC^+^ T cells based on the expression of killer cell lectin-like receptor G1 (KLRG1), and lack of expression of CD27 and CD28 (**Figure 12A**). Select previously validated metabolic antibodies (29) were used to characterize metabolic proteins/enzymes in CGC^+^ CD4^+^ and CGC^+^ CD8^+^ T cells. These included metabolic proteins involved in fatty acid metabolism (CPT1A), the tricarboxylic acid cycle and the electron transfer chain (ATP synthase (ATP5A)), mitochondrial expression (voltage-dependent ion channel 1 (VDAC1)), glycolysis and fermentation (hexokinase 2 (HK2)), signaling and transcription (ribosomal protein S6, pS6)) and amino acid metabolism (CD98) (**Figure 12B**). Of all the metabolic proteins tested, CPT1A expression was higher on CGC^+^ CD4^+^ T cells than all other CD4 subsets (**Figure 12C**), and when compared to all other non-CGC^+^ CD4^+^ T cells, both CPT1A and CD98 were higher while pS6 was lower (**Figure 12D**). CGC^+^ CD8^+^ T cells also had higher CPT1A than other CD8^+^ T cell subsets, while pS6 was significantly lower (**Figures 12E, F**). Taken together, higher levels of CPT1A in both CGC^+^ CD4^+^ and CGC^+^ CD8^+^ T cells implicate FAO as a potential source of energy in CGC^+^ T cells. Lower pS6 compared to other T cells may indicate that CGC^+^ cells have a lower basal level of translation than other memory subsets.

## Discussion

In PWH, systemic inflammation remains chronically elevated despite consistent suppression of plasma viremia on ART and reconstitution of total CD4^+^ T cells. CMV co-infection contributes to inflammation by engaging cells from both the innate and adaptive immune arms (52, 53). Importantly, latent CMV infection permanently alters the immune repertoire in aging individuals regardless of their HIV status, inducing a unique T cell response characterized by an increase in TEM and TEMRA cells (9, 54, 55). While doing so, CMV alters the responses to other viruses such as the impaired CD8^+^ T cell response to Epstein Barr virus (11). In PWH, the memory CD4^+^ T cell pool against CMV is significantly inflated, but the exact mechanism is unknown (13, 56). It has been proposed that higher CMV viral replication at the tissue level may be an important driver of this T cell expansion; however, the tissue(s) responsible for the inflation of T cell responses has not been identified (10). To date, we lack a clear mechanism that explains the role of CMV in the pathogenesis of cardiometabolic diseases.

Unlike the innate immune system, cells of the adaptive immune system appear more prone to influence by environmental and physiologic factors (54). To our knowledge, this is the first study to show a correlation between CGC^+^ CD4^+^ T cells and plasma metabolites, including several amino acid and carbohydrate metabolism pathways. Notably, despite the modest relationship between CGC^+^ CD4^+^ T cells and fasting blood glucose or starch metabolites, unstimulated CGC^+^CD4^+^ T cells exhibited low glucose and high mitochondrial dependence ex-vivo. This was also observed in primary CGC^+^ CD4^+^ T cells expanded ex-vivo. Hence, the relationship between CGC^+^ CD4^+^ T cells and fasting blood glucose does not appear to stem from higher glucose dependence. While circulating CGC^+^ CD8^+^ and CGC^+^ CD4^+^ T cells were strongly correlated, they differed in their relationships with CAC, NAFLD, diabetes, and pericardial fat volume. This suggests that although CGC^+^ CD4^+^ and CGC^+^ CD8^+^ T cells are likely related by their response to CMV antigens, and the circulating proportion of both increases with progressive glucose intolerance, the role or interaction of these cells with the processes contributing to end-organ disease may differ.

CMV-specific T cells can be identified using tetramers, or by the functional expression of inflammatory cytokines after exposure to antigen-presenting cells infected with CMV or pulsed with CMV peptides (13, 57, 58). Other surface marker combinations including CX3CR1, CD57, KLRG1, and a lack of expression of CD28, have also been used to define CD4^+^ and CD8^+^ T cell subsets associated with CMV seropositivity (27, 59–63). However, technical challenges to defining T cell responses to CMV include the size of its genome and HLA restriction of T cell responses. In this study, we showed that CMV-specific T cells in individuals with HIV were predominantly CGC^+^. Further studies are underway to define the breadth of TCRs that are recognized by the CGC^+^ subset of CD4^+^ T cells among PWH and HIV-negative individuals, to further understand the extent to which these cells react to CMV epitopes.

CD4^+^ CD28^-^ T cells are a subset of cytotoxic cells that are increased in autoimmune diseases and with persistent infections such as HIV (64, 65). Specifically, high proportions of CD4^+^ CD28^-^ T cells have been reported in individuals with unstable angina (66), within unstable plaques (67), and in persons with recurrent coronary events (68). Our group has previously shown that CD4^+^ CD28^-^ T cells are also increased prior to the development of incident diabetes in PWH (45). Although CD4^+^ CD28^-^ T cells are oligoclonal (67), different antigens may stimulate CD4^+^ CD28^-^ T cells including HIV and CMV. However, studies in which treatment of individuals with anti-CMV therapies has reduced the proportion of CD4^+^ CD28^-^ T cells in circulation suggest that targeting the CMV viral burden may be a feasible therapeutic approach (27).

**Figure 13:**
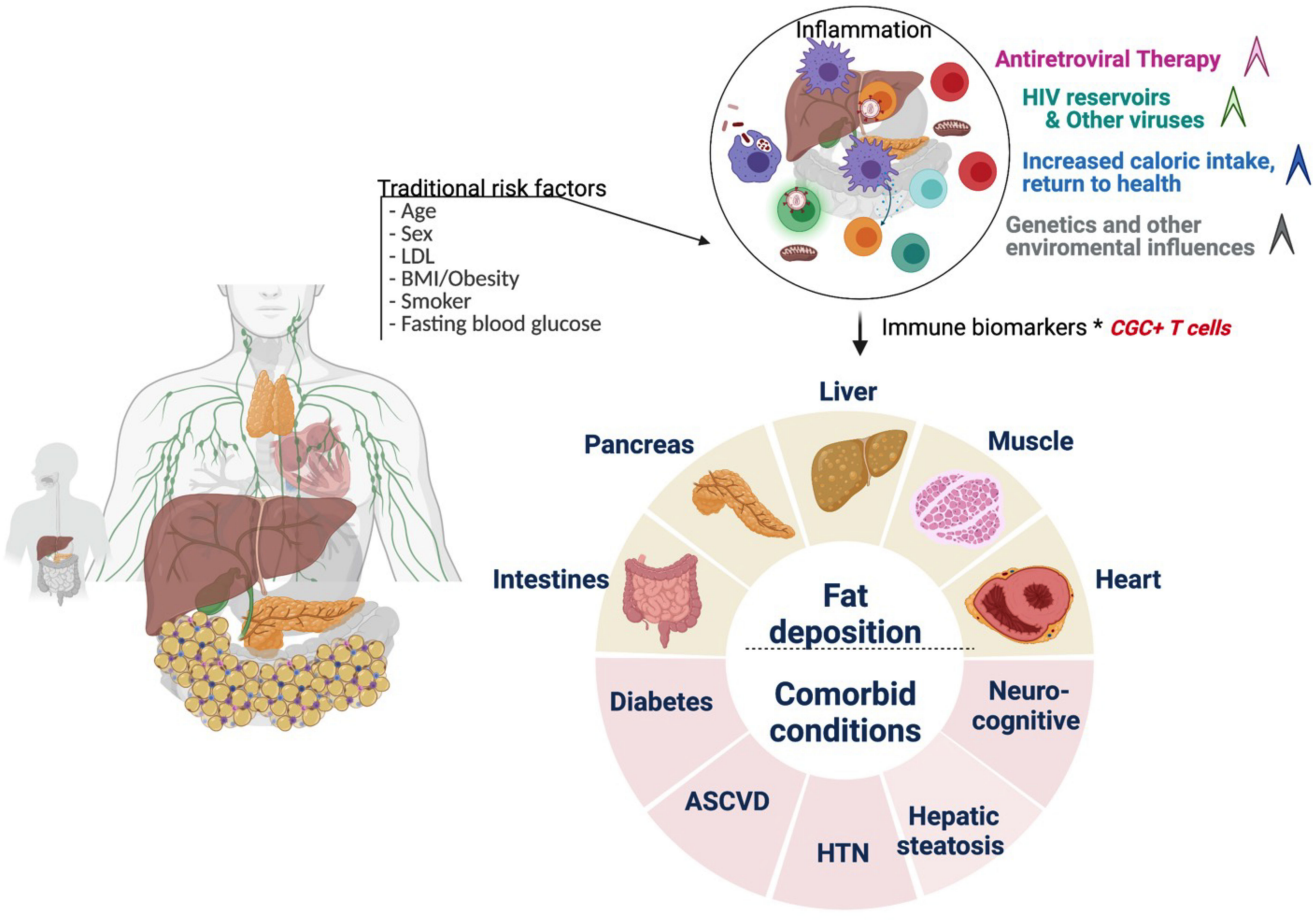
Conceptual Model. Traditional cardiovascular risk factors (age, sex, low density lipoprotein, body mass index, smoking status, and fasting blood glucose) and HIV-associated factors antiretroviral therapy, viral load, and CD4^+^ T cell count) may contribute to inflammation that drives cardiometabolic disease. CGC^+^ CD4^+^ T cells are largely CMV-specific T cells that are inflated in PWH and may have a diagnostic and mechanistic role in the pathogenesis of cardiometabolic disease. Figure created using Biorender.

Although our study suggests a role for CGC^+^ CD4^+^ T cells in the pathogenesis of cardiometabolic disease in PWH, the cross-sectional design precludes an assessment of causality. Following longitudinal cohorts to correlate changes between frequencies of CGC^+^ CD4^+^ T cells and cardiometabolic clinical endpoints may define a threshold to classify a subset of persons in which residual inflammation from CMV is a risk factor for the development of comorbidities. Second, our study did not include matched controls without HIV and with metabolic diseases (non-diabetic, pre-diabetic, and diabetic), which would be necessary to understand whether CGC^+^ CD4+ T cells demonstrate a similar role in HIV-negative individuals. Third, the mechanism by which CGC^+^ T cells could alter metabolic health is an area of ongoing investigation. Further studies are underway by our group to understand how CGC^+^ CD4^+^ T cells impact the pathogenesis of cardiometabolic diseases given their cytotoxic capacity, particularly within adipose tissue and vascular structures (17). One intriguing possibility is that these cells could be stimulated by non-viral antigens that mimic CMV epitopes and contribute to the development of metabolic disease. The model figure (**Figure 13**) summarizes the traditional and HIV-specific risk factors that contribute to ectopic fat distribution and the increased prevalence of cardiometabolic disease in PWH. The role of CGC^+^ cells in cardiometabolic disease risk stratification and its potential role in identifying individuals with a higher risk of developing comorbidities needs to be explored in detail. Eventually, treating CMV or targeting CGC^+^CD4^+^ T cells may provide a target that can improve outcomes in PWH and possibly extend to a subset of individuals in the general population.

## Data availability statement

The original contributions presented in the study are included in the article/**Supplementary Material**. Further inquiries can be directed to the corresponding author.

## Ethics statement

The studies involving human participants were reviewed and approved by Vanderbilt University Institutional Review Board. The patients/participants provided their written informed consent to participate in this study.

## Author contributions

CNW and JK contributed to the conception of the study. CNW, CG, HF, DG, JO, EN, SK, SM, JK contributed to the design of the study. CG, HF, DG, JS, CMW, JO, RG, SPa, SPr contributed to data collection and analysis. SG, DS, DH, MM, SB, TT, SK, SM, JK contributed to the integration of concepts. CNW performed the statistical analysis. CNW and JK wrote the first draft of the manuscript. All authors contributed to manuscript revision, read, and approved the submitted version.
