# Supplementary material for: CD4+ T cells expressing CX3CR1, GPR56, with variable CD57 are associated with cardiometabolic diseases in persons with HIV
Source: Front Immunol. 2023 Feb 14;14:1099356. doi: 10.3389/fimmu.2023.1099356 (PMC9971959; doi:10.3389/fimmu.2023.1099356)
Supplement: Supplementary file 1 [file DataSheet_1.pdf]

CD4$^+$ T cells Expressing CX3CR1, GPR56, with variable CD57 are Associated with Cardiometabolic Disease in Persons with HIV

Authors: Celestine N. Wanjalla[1†], Curtis L. Gabriel[2], Hubaida Fuseini[1], Samuel S. Bailin[1], Mona Mashayekhi[4], Joshua Simmons[1], Christopher M. Warren[1], David R. Glass[3], Jared Oakes[1], Rama Gangula[1], Erin Wilfong[5,6], Stephen Priest[1], Tecla Temu[7], Evan W. Newell[3], Suman Pakala[1], Spyros A. Kalams[1], Sara Gianella[8], David Smith[8], David G. Harrison[9], Simon A. Mallal[1], John R. Koethe[1,10]

[†] Corresponding author: Celestine N. Wanjalla, MD, Ph.D.; Division of Infectious Diseases, Vanderbilt University Medical Center, A-2200 MCN, 1161 21st Ave S., Nashville, TN, 37232-2582. (615) 322-2035 (o), (615) 343-6160 (f), celestine.wanjalla@vumc.org

Supplemental Table 1. REAGENTS

CYTOF Reagents

**Target**	**Metal Tag**	**Tag Isotope**	**Source or reference**
CD57	Neodymium (Nd)	142	Fluidigm
CD4	Neodymium (Nd)	145	Fluidigm
CD8a	Neodymium (Nd)	146	Fluidigm
GPR56	Samarium(Sm)	154	CIC core
OX40	Gadolinium (Gd)	158	Fluidigm
PD-1	Gadolinium (Gd)	155	Fluidigm
CD28	Gadolinium (Gd)	160	Fluidigm
CD27	Erbium (Er)	167	Fluidigm
CX3CR1	Ytterbium (Yb)	172	CIC core
Cisplatin	Platinum (Pt)	195	Fluidigm
Nuc acid --Ir	Iridium (Ir)	191/193	Fluidigm
CD45	Yttrium (Y)	89	Fluidigm
DNA	Rhodium (Rh)	103	Fluidigm
CD45	Cadmium (Cd)	106	Fluidigm
CD45	Cadmium (Cd)	110	Fluidigm
CD45	Cadmium (Cd)	111	Fluidigm
CD8	Cadmium (Cd)	112	BioLegend
CD14	Cadmium (Cd)	113	BioLegend
CD45	Cadmium (Cd)	114	Fluidigm
CD57	Indium (In)	115	BioLegend
CD45	Cadmium (Cd)	116	Fluidigm
CD45RA	Neodymium (Nd)	145	BioLegend
CD4	Neodymium (Nd)	155	BioLegend
VDAC1	Terbium (Tb)	159	abcam

CCR7	Erbium (Er)	168	BioLegend
CD28	Ytterbium (Yb)	173	BioLegend
ATP5A	Ytterbium (Yb)	174	abcam
pS6	Lutetium (Lu)	175	Fluidigm
CPT1A	Ytterbium (Yb)	176	abcam
Viability	Platinum (Pt)	195	Fluidigm
KLRG1	Platinum (Pt)	196	eBioscience
CD98	Platinum (Pt)	198	BD Biosciences
CD3	Bismuth (Bi)	209	BioLegend
**Flow Cytometry Reagents**			
**Target**	**Fluorescent Tag**	**Source or Reference**	**Identifier**
**Panel 1**			
CD3 (Clone SK7)	BV786	BD Biosciences	#563800
CD4 (Clone RPA-T4)	PcPCy5.5	BD Biosciences	#560650
CD8 (Clone PRA-T8)	A700	BD Biosciences	#557945
CCR7 (Clone 150503)	BV421	BD Biosciences	#562555
CD45RO (Clone UCHL1)	PECF594	BD Biosciences	#562299
LIVE/DEAD Fixable Aqua	N/A	ThermoFisher	L34957
TETRAMERS	APC	NIH Emory Core	N/A
CD57 (Clone NK-1)	FITC	BD Pharmingen	# 555619
CX3CR1(Clone 2A9-1)	PE	BD Biosciences	#565796
GPR56 (Clone CG4)	PECY7	BioLegend	#358205
CD14 (Clone M5E2)	V500	BD Biosciences	#561391
CD19 (Clone HIB19)	V500	BD Biosciences	#561121
Panel 2 (Cytek)			

CD3 (Clone UCHT1)	Nova Blue 660-120	Invitrogen	#H002T02B08
CD4 (Clone RPA-T4)	BV650	BioLegend	#356935
CD8 (Clone PRA-T8)	BV570	BioLegend	#301037
CCR7 (Clone G043H7)	Spark NIR 685	BioLegend	#353257
CD45RO (Clone UCHL1)	PE-CF594	BD Biosciences	#562299
LIVE/DEAD Fixable Aqua	Zombie Violet	BioLegend	#423113
CD57 (Clone NK-1)	FITC	BD Biosciences	#555619
CX3CR1(Clone 2A9-1)	PE	BD Biosciences	#565796
GPR56 (Clone 4C3)	APC	BioLegend	#391905
KLRG1 (Clone 2F1-KLRG1)	APC Cy7	BioLegend	#138425
CD38 (Clone HIT2)	AF700	BD Biosciences	#560678
CXCR3 (Clone G025H7)	PE-CY5	BioLegend	#353755
PD1 (Clone EH12.2H7)	PE-CY7	BioLegend	#329918
CD27 (Clone O323)	PE Fire 810	BioLegend	#302859
CD28 (Clone CD28.2)	BV711	BioLegend	#302947
CD14 (Clone 63D3)	APC Fire 810	BioLegend	#367155
CXCR5 (Clone J252D4)	BV785	BioLegend	#356935
Anti-puromycin (Clone 12D10)	AF488	Millipore Sigma	MABE343
Puromycin solution	N/A		
Oligomycin A	N/A	Sigma Aldrich	75351-5MG
2-deoxy-D-glucose 2mM	N/A	Sigma Aldrich	D8375
Foxp3 / Transcription Factor Staining Buffer Kit	N/A	TonBio	TNB-0607-KIT

Supplemental Table 2. HLA of selected participants with tetramer analysis

Participant	Study ID	HLA A	HLA B	HLA C	HLA DQB1	HLA DRB1
**1**	**3024**	**02:01**	*Not Available*	*Not Available*	*Not Available*	*Not Available*
**2**	**10038**	**02:01 + 02:01**	27:03 + 44:02	01:02 + 05:01	*Not Available*	**07:01** + 13:01
**3**	**10114**	**02:01 + 02:01**	18:01 + 44:02	05:01 + 07:01	*Not Available*	04:01 + 11:04
**4**	**10064**	**02:01** + 11:01	37:0 +. 44:02	05:01 + 06:02	*Not Available*	**07:01** + 11:03
**5**	**1111**	**02:01 + 68:01**	08:01 + 51:01	07:01 + 16:01	02:02 + **06:02**	**07:01 + 11:01**
**6**	**10069**	01:01 + 02:53	53:01 + 57:01	04:01 + 07:01	*Not Available*	**07:01** + 08:04
**7**	**10027**	01:01 + **02:01**	08:01 + 57:01	06:02 + 07:01	*Not Available*	**03:01 + 07:01**
**8**	**10042**	03:01 + 03:01	14:02 + 40:01	03:04 + 08:02	*Not Available*	01:01 + 13:02
**9**	**10074**	01:01 + 26:01	08:01 + 57:01	06:02 + 07:01	*Not Available*	03:01 + 04:04
**10**	**10122**	30:02 + 68:01	57:03 + 58:0	02:02 + 07:01	*Not Available*	**07:01 + 07:01**
**11**	**1129**	**02:01** + 68:02	07:02 + 07:02	07:02 + 07:02	**06:02 + 06:02**	15:01 + 15:03

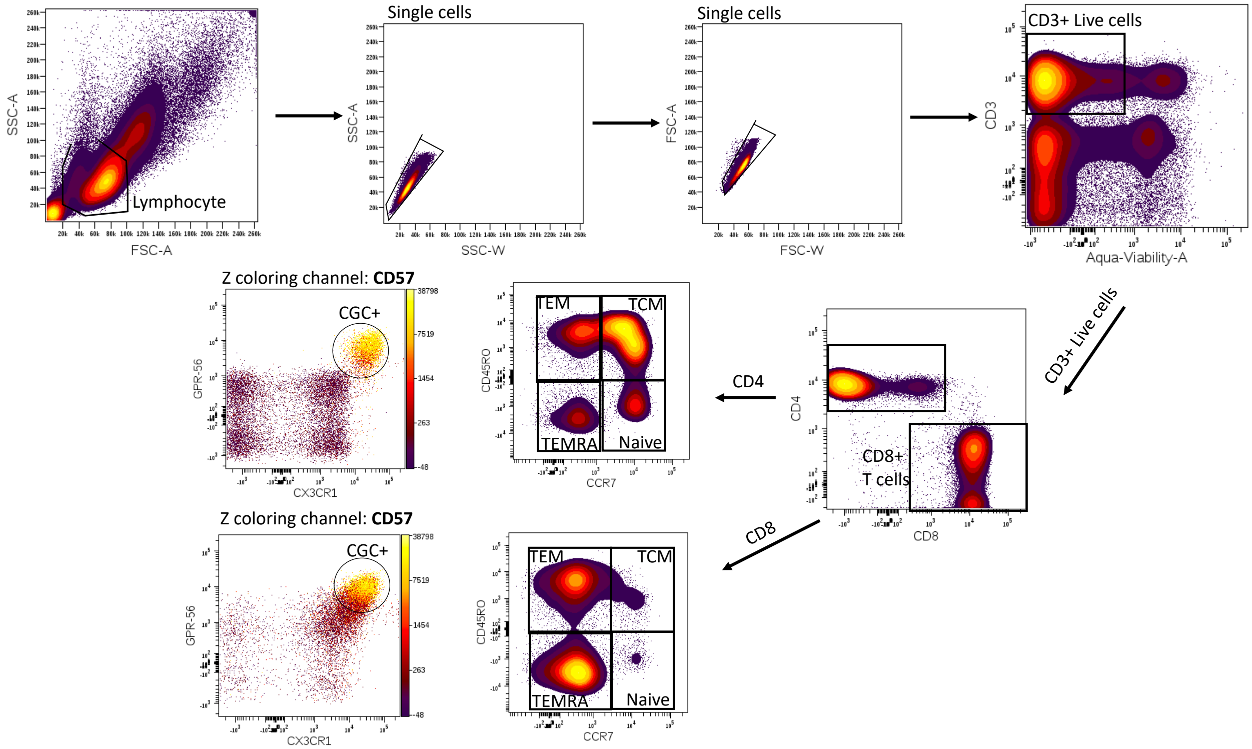

Supplemental Figure 1. Flow cytometry gating strategy. Two-dimensional dot plots show the gating strategy used to define lymphocytes, excluding doublets. We excluded dead cells using a viability dye while gating for $CD3^{+}$ T cells. $CD4^{+}$ and $CD8^{+}$ T cells are gated on $CD3^{+}$ Live cells. Memory subsets are defined using CCR7 and CD45RO. We have also included a plot showing CX3CR1 and GPR56 expression on $CD4^{+}$ and $CD8^{+}$ T cells. CD57 expression on the $CX3CR1^{+}$ and $GPR56^{+}$ cells is depicted using the Z coloring channel. Note that for sorted cells, we sort the top right corner of $CX3CR1^{+}$ and $GPR56^{+}$ cells. Data were collected using a BD FACS Aria II and analysis was performed using Cytobank. A similar gating strategy was used to gate cells that were run on the Cytek.

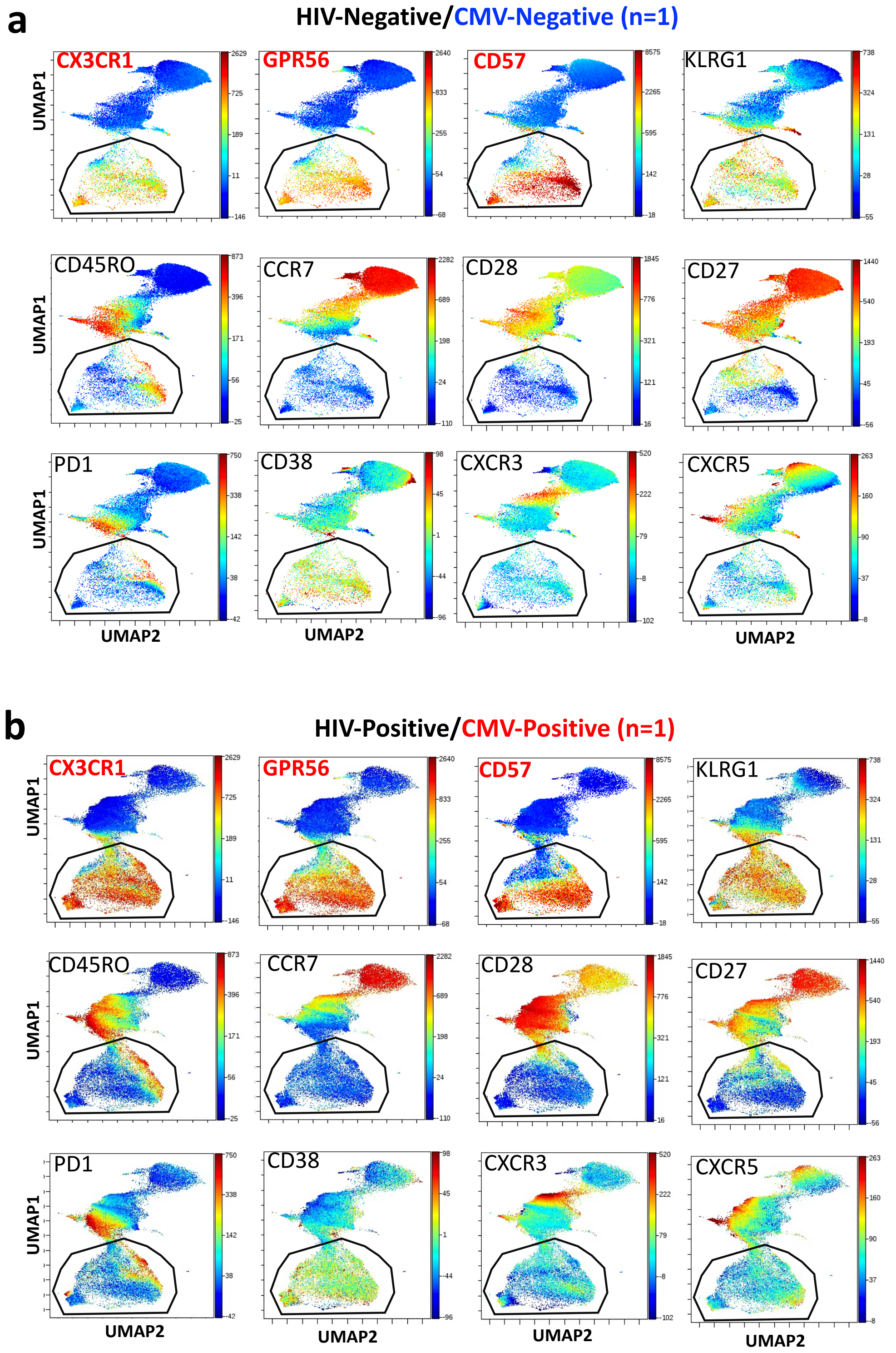

Supplemental Figure 2. CGC$^+$ CD8$^+$ T cells are KLRG1$^+$, CD38$^+$, CD28$^-$, CD27$^-$, and PD1$^{+/-}$. Representative UMAPs were generated from PBMCs of a CMV-negative/HIV-negative donor (a) and a CMV-positive HIV-positive donor (b). Each plot has a marker that is highlighted on the z-coloring channel.

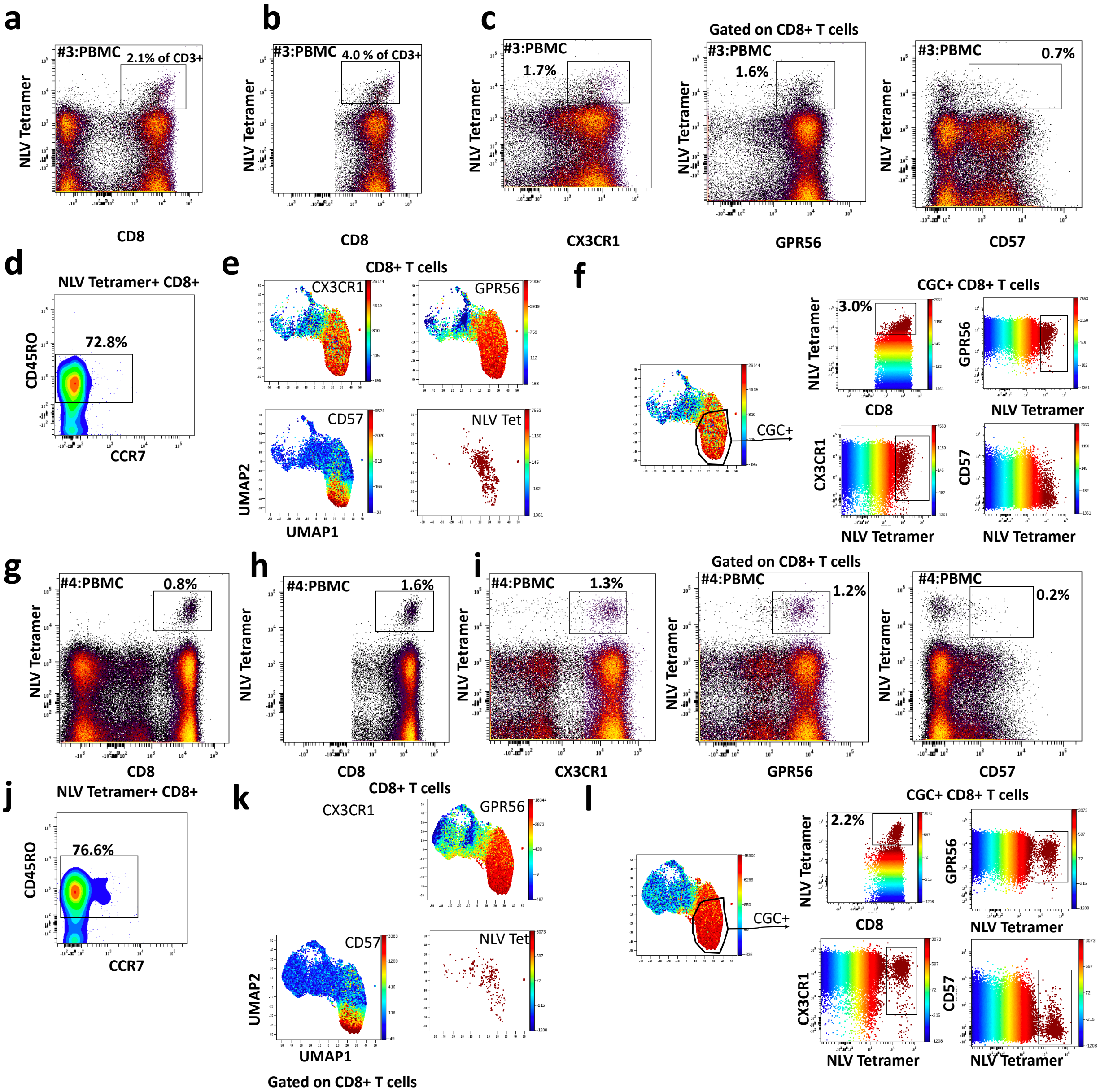

Supplemental Figure 3. NLV-specific CD8$^+$ T cells are CX3CR1$^+$, and GPR56$^+$ with variable expression of CD57 (in 2 additional participants). Peripheral blood CD8$^+$ T cells with TCRs that recognize the NLV epitope were evaluated as a proportion of total CD3$^+$ T cells and total CD8$^+$ T cells in two additional PWH (a-b; g-h). Two-dimensional plots show the expression of CX3CR1, GPR56, and CD57 by NLV-specific CD8$^+$ T cells (c, i). Memory cell phenotypes were classified as TEM (CD45RO$^+$ CCR7$^-$) and TEMRA (CD45RO$^-$ CCR7$^-$) (d, j). UMAP of CD8$^+$ T cells showing the NLV tetramer$^+$ CD8$^+$ T cells among the CGC cells (e,k). We gated on the CGC$^+$ cluster and show the proportion of tetramer-positive cells among the CGC$^+$ cell cluster, highlighting NLV-tetramer$^+$ cells using the Z color channel (f,l).

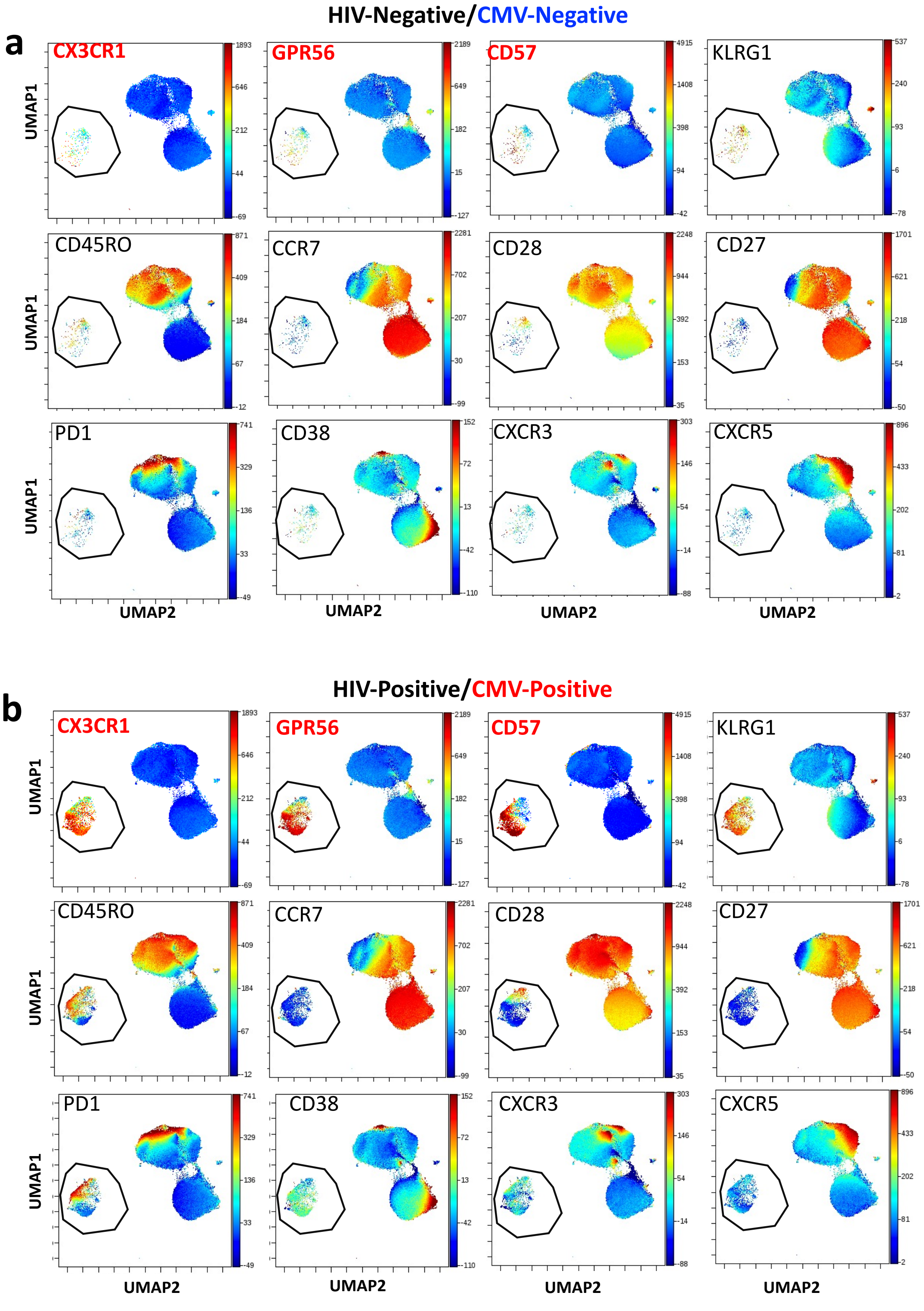

Supplemental Figure 4. CGC$^+$ CD4$^+$ T cells are KLRG1$^+$, CD38$^{+/-}$, PD1$^{+/-}$, CD28$^-$, CD27$^-$. Representative UMAPs were generated from PBMCs of a CMV-negative/HIV-negative donor (a) and a CMV-positive HIV-positive donor (b). Each plot has a marker that is highlighted on the z-coloring channel.

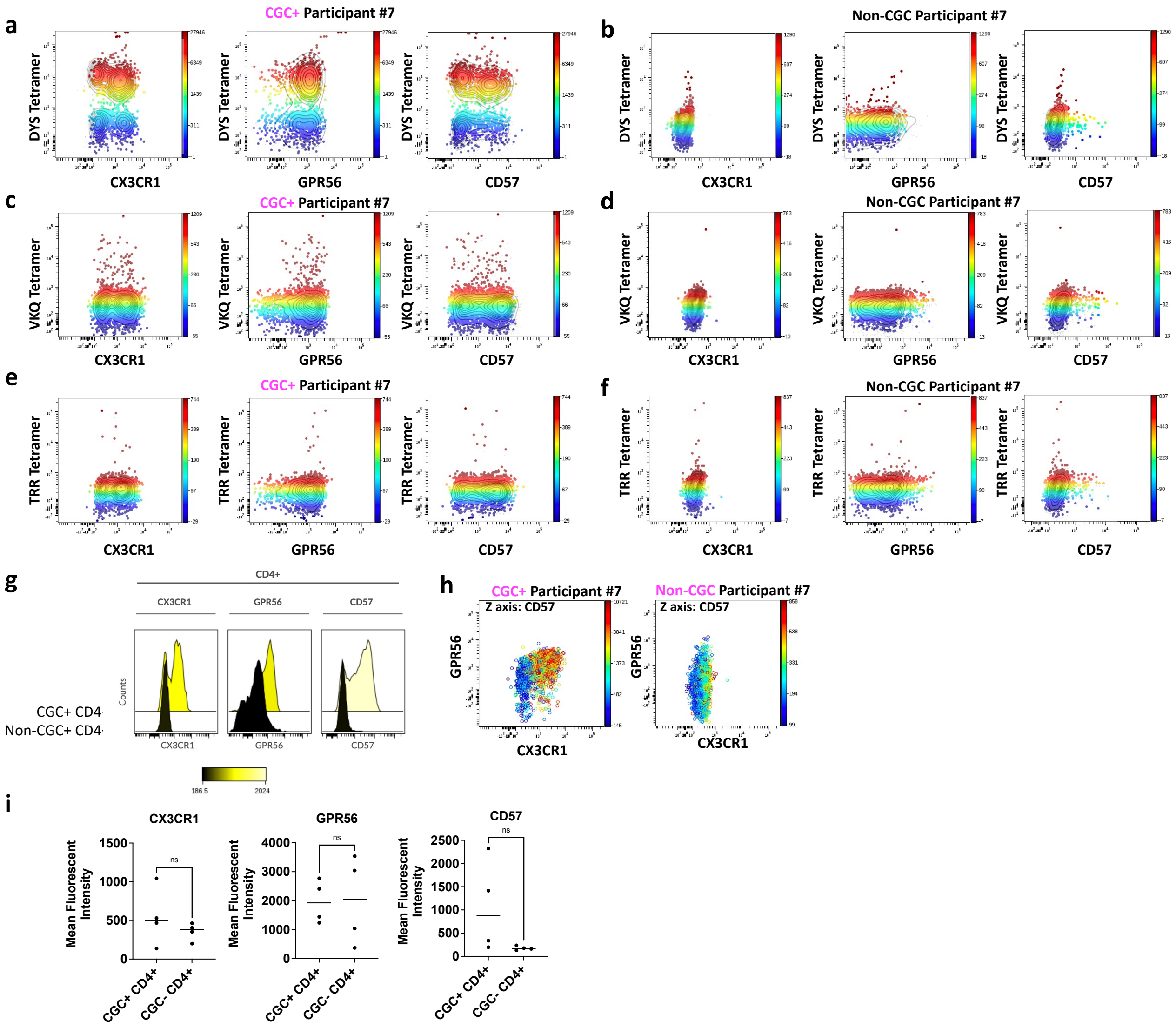

Supplemental Figure 5. CX3CR1, GPR56, and CD57 expression on expanded CMV-tetramer specific CD4^{+} T cells. Two-dimensional flow cytometry plots showing expression of the tetramer, CX3CR1, GPR56, and CD57 on expanded CGC^{+} CD4^{+} (a,c,e) and non-CGC^{+} CD4^{+} (b,d,f) T cells from participant #7. Histograms show CX3CR1, GPR56, and CD57 on CGC^{+} and non-CGC^{+} CD4^{+} T cells (g), and two-dimensional flow plots show differences in CX3CR1/GPR56 with z-axis color channel showing CD57 expression (h). Mean fluorescence intensity (MFI) of CX3CR1, GPR56, and CD57 on matched tetramer^{+} (DYS, TRR, and VKQ) T cells on ex-vivo expanded CGC^{+} and non-CGC^{+} CD4^{+} T cells (i).

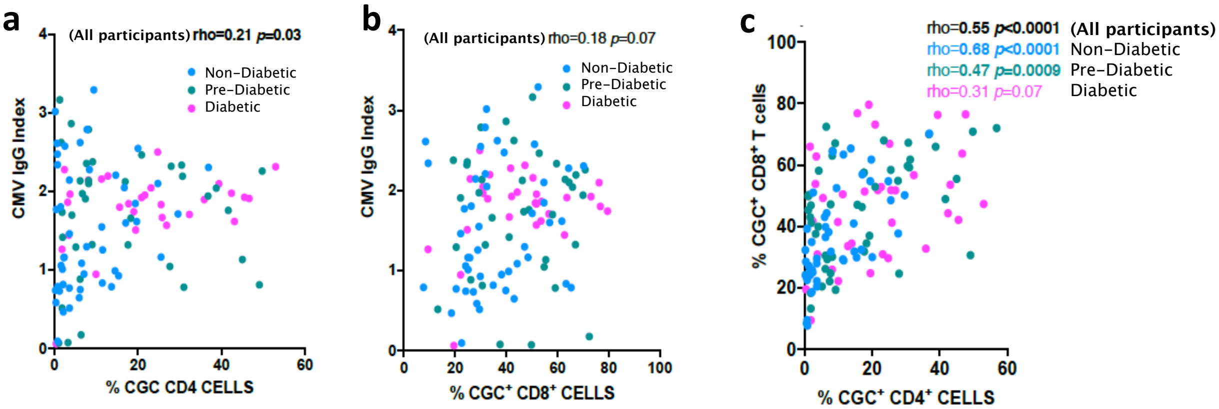

Supplemental Figure 6. CGC$^+$ CD4$^+$ and CD8$^+$ T cells are correlated with CMV antibody titers. Correlation plot showing relationships between CMV IgG plasma titers with CGC$^+$ CD4$^+$ (a) and CGC$^+$ CD8$^+$ T cells (b). Correlation analysis was also performed between CGC$^+$ CD4$^+$ T cells and CGC$^+$ CD8$^+$ T cells (c). Statistical analysis by Spearman's rank correlation analysis.

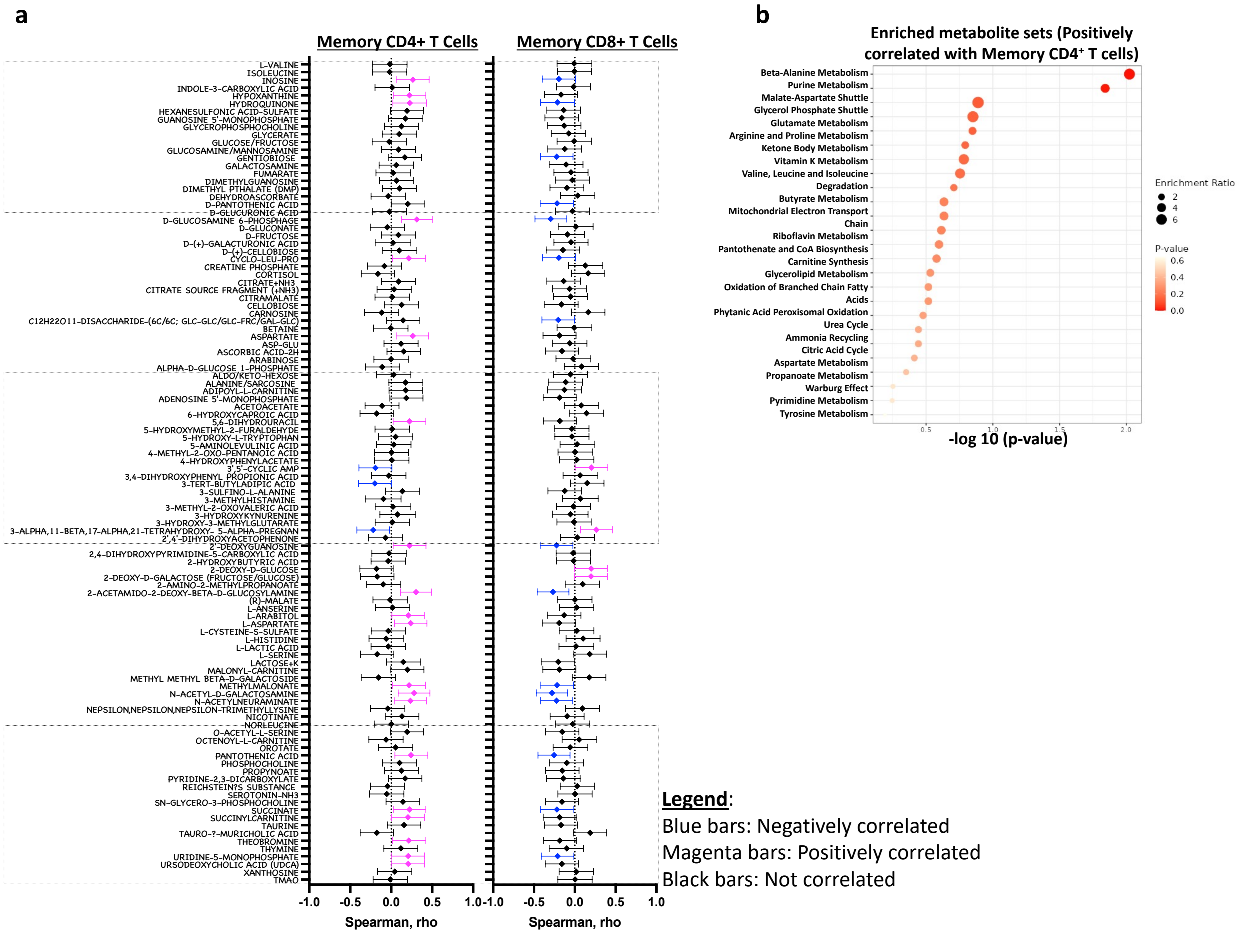

Supplemental Figure 7. Total memory CD4^{+} and CD8^{+} T cells are not consistently correlated with plasma metabolites. Forest plots show Spearman's rank correlation coefficients between plasma metabolites and the proportion of total memory CD4^{+} T cells (left panel) and total memory CD8^{+} T cells (right panel) (a). The top twenty-five metabolite sets in the enrichment analysis (number of metabolites/expected metabolites per set) were performed with metabolites that were positively correlated with memory CD4^{+} T cells (b). There were not enough metabolites positively correlated with memory CD8^{+} T cells to perform the same analysis. Statistical analysis by Spearman's rank correlation. Color code: Blue, negative correlation $p<0.05$; Magenta, positive correlation $p>0.05$; black, insignificant correlation. Over Representation Analysis (ORA) of plasma metabolites was performed using MetaboAnalyst 5.0 with the hypergeometric test. One-tailed adjusted p-values are provided after correcting for multiple testing using false discovery rate (FDR).

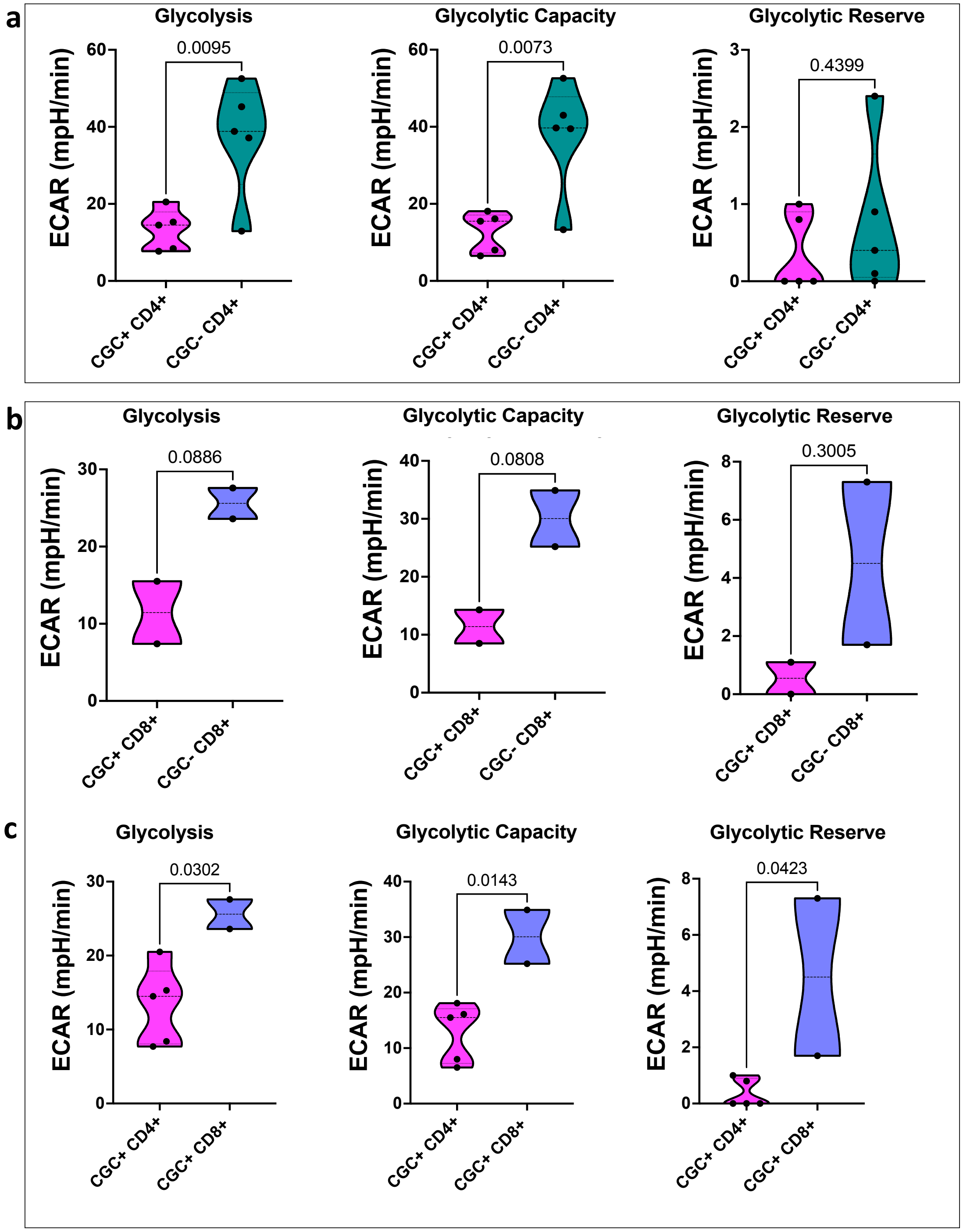

Supplemental Figure 8. CGC⁺ T cells expanded using CD3/CD28 beads do not engage in glycolysis as effectively as non-CGC⁺ T cells. (a) ECAR assay showing differences in glycolysis, glycolytic capacity, and glycolytic reserve among CGC⁺ and non-CGC⁺ CD4⁺ T cells; (b-c) CGC⁺ and non-CGC⁺ CD8⁺ T cells and (d) CGC⁺ CD4⁺ and CD8⁺ T cells. Representative of two independent experiments with expanded CGC cell lines. This experiment was done using cells expanded from 5 different participants (representative of two repeat experiments). Statistical analysis by t-test.

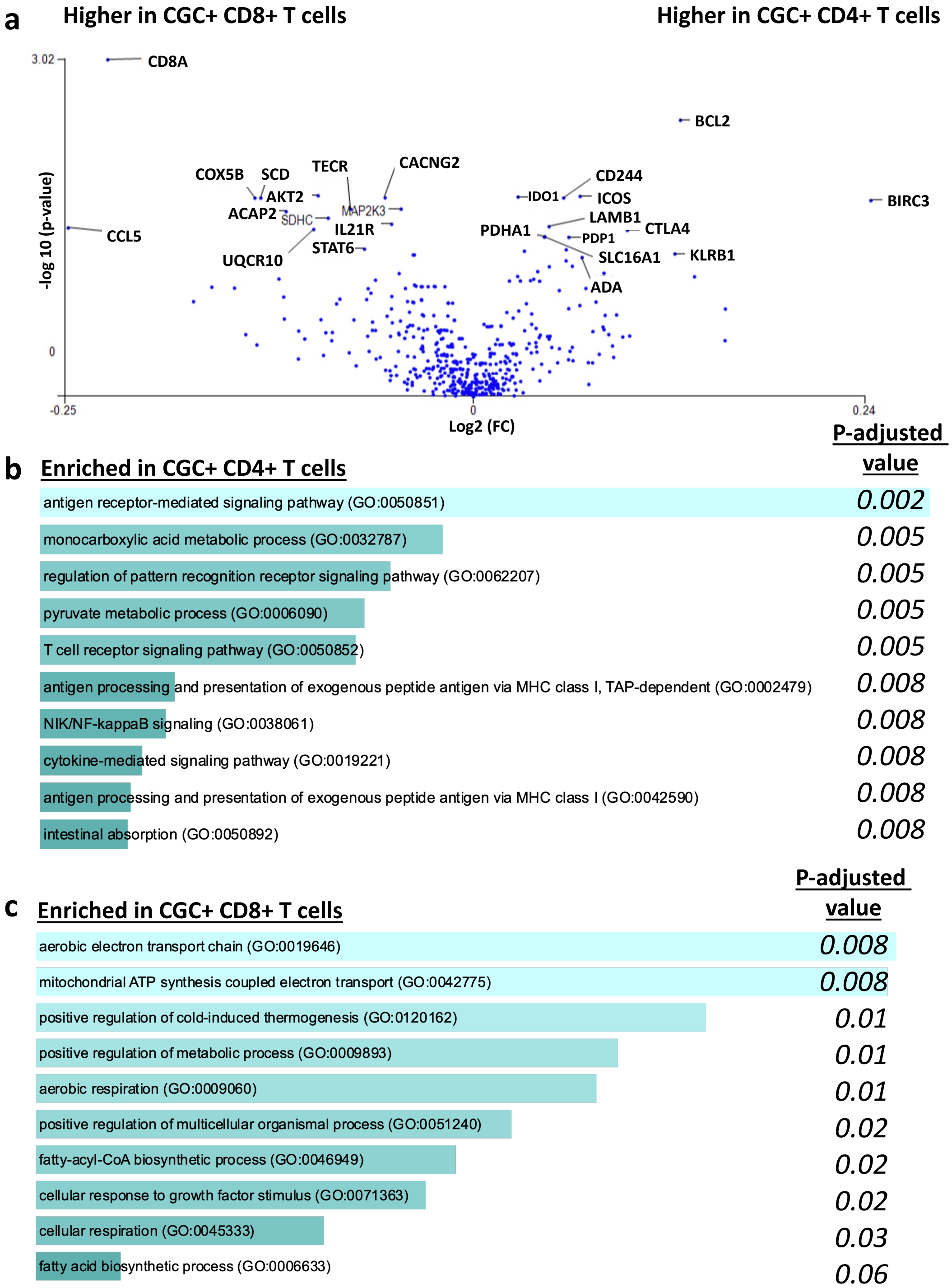

Supplemental Figure 9. CGC$^+$ CD8$^+$ T cells are enriched for genes in the aerobic transport chain. Volcano plot showing differential metabolic gene expression between CGC$^+$ CD4$^+$ T cells and CGC$^+$ CD8$^+$ T cells (a). Differential gene expression was performed on VGAS using the Kruskal-Wallis method with BH for multiple corrections. GO biological processes of differentially expressed genes higher in CGC$^+$ CD4$^+$ (b) and CGC$^+$ CD8$^+$ (c) are automatically identified with the Leiden algorithm with a p-value < 0.05 from the top 10 enriched pathways shown. The p-value is computed using Fisher's exact test and the correction of multiple testing by Benjamini-Hochberg.
